# Comparative analysis of prokaryotic communities, hydrography, and biogeochemistry in Atlantic vs non-Atlantic influenced Svalbard fjords

**DOI:** 10.1186/s12866-026-04821-2

**Published:** 2026-02-27

**Authors:** Joana Costa, Francisco Pascoal, Mafalda S. Baptista, Haakon Hop, Philipp Assmy, Anette Wold, Catarina Magalhães, Pedro Duarte

**Affiliations:** 1 Interdisciplinary Centre of Marine and Environmental Research, University of Porto, Terminal de Cruzeiros do Porto de Leixões, Av. General Norton de Matos s/n, Porto, 4450-208 Portugal; 2 Departamento de Biologia, Faculdade de Ciências, Universidade do Porto, Rua do Campo Alegre s/n, Porto, 4169–007 Portugal; 3 Norwegian Polar Institute, Fram Centre, Tromsø, N-9296 Norway

**Keywords:** Arctic fjords, Water masses, Atlantification, Biogeochemistry, Polar microbiology, Prokaryotic communities, Nitrogen cycle

## Abstract

**Background:**

Fjords in Svalbard are undergoing significant changes due to climate warming. Those along the west coast of Spitsbergen are particularly affected by the increasing influence of “warm” Atlantic Water (AW), a process known as Atlantification. We compared Kongsfjorden, a relatively “warm” fjord on the west coast, with Rijpfjorden, a typical cold Arctic fjord on the north coast of Nordaustlandet, combining physical and biogeochemical data with 16S rRNA gene amplicon and shotgun metagenomic sequencing. We hypothesize that differences in fjords’ water masses and prokaryotic communities provide insight into the effects of Atlantification as it expands eastwards along the shelf north of Svalbard.

**Results:**

We found that warm AW dominated in Kongsfjorden, whereas Rijpfjorden was dominated by cold Arctic Water and Winter Cooled Water. Our results suggest that the Atlantic-influenced Kongsfjorden is a nutrient sink, whereas Rijpfjorden showed similar behavior only in 2016, a particularly warm year, otherwise no clear sink/source role could be identified. Analysis of 16S rRNA gene sequences revealed that Proteobacteria had higher relative abundances in Kongsfjorden while Bacteroidota dominated in Rijpfjorden. Ammonium and nitrite-oxidizing prokaryotes were most prevalent in deeper water masses of both fjords. The archaeal taxa of the ammonia-oxidizing community, mainly *Nitrosopumilus* and *Nitrosopelagicus,* were consistently more dominant than ammonium and nitrite-oxidizing bacteria. Denitrification and nitrogen fixation genes differed between the fjords, with Kongsfjorden having a higher coverage of diazotroph genes.

**Conclusions:**

Kongsfjorden and Rijpfjorden displayed distinct hydrographic conditions, with Kongsfjorden being under a stronger influence of Atlantification. Our results suggest that warmer water masses are linked to higher nutrient uptake. The clear association between microbial communities and water masses offers insight into changes driven by Atlantification.

**Supplementary Information:**

The online version contains supplementary material available at 10.1186/s12866-026-04821-2.

## Introduction

Arctic surface air temperature has increased three to four times faster than the global mean over the last 50 years, as a result of Arctic Amplification [1, 2], resulting in a drastic decrease in Arctic summer sea-ice extent and thickness [3]. The loss of the ice pack creates a positive feedback loop due to increased absorption of heat by the darker ocean and consequent warming of the Arctic Ocean known as the climate-albedo feedback. The retreat of the sea ice in the Arctic has also been associated with the expansion of Atlantic Water (AW) into the Arctic Basin, a process termed Atlantification [4, 5]. Moreover, AW has been warming over the last ~ 100 years and the highest temperatures in the last 2000 years were observed in the twenty-first century [6–8]. This warming of AW reduces its density and facilitates its mixing with the cold and lower-salinity Polar Surface Water (sensu [9]), increasing the transfer of heat and nutrients to the surface, sea-ice melting and, possibly, primary production. Atlantification has been spreading towards the east along the northern Svalbard shelf [4, 10].

**Fig. 1:**
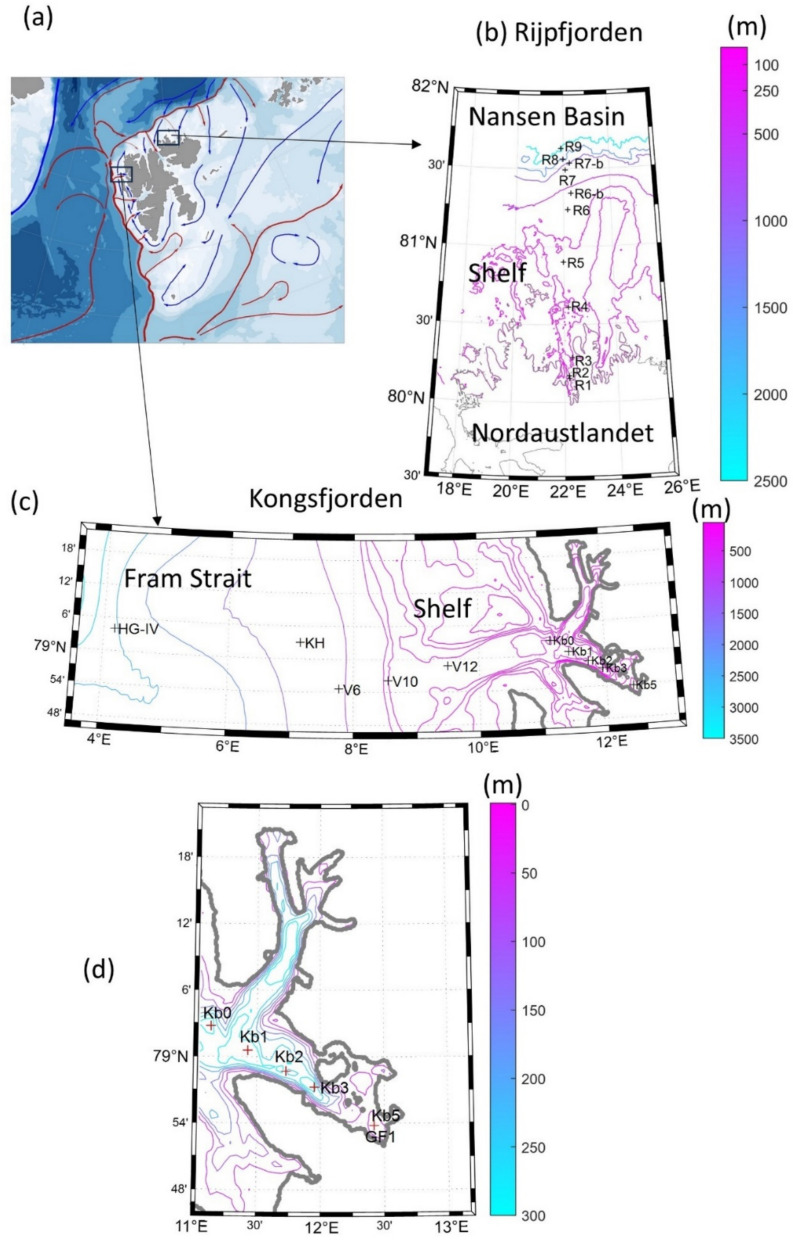
**a** Geographical context of the study sites, Rijpfjorden located in Nordaustlandet (NE Svalbard) and Kongsfjorden in West Spitsbergen (W Svalbard). The West Spitsbergen Current is shown in red and the Spitsbergen Polar Current in blue. This panel was produced with the PlotSvalbard R package version 0.9.2 [14]. **b**, **c** Rijpfjorden and Kongsfjorden bathymetry and location of sampling sites, respectively. **d** Same as (**c**) but only for the inner part of Kongsfjorden. Bathymetry was downloaded from https://www.gebco.net/data_and_products/gridded_bathymetry_data/arctic_ocean/ (accessed 14.02.2026) at 200 m × 200 m grid-cell spacing

Fram Strait, between Greenland and the Svalbard archipelago, is the main gateway between the Atlantic and the Arctic Ocean. The hydrography of the fjords located along the west coast of Svalbard (Spitsbergen) is influenced by cold and fresh Arctic Water (ArW), carried by the Spitsbergen Polar Current (SPC) on the West Spitsbergen Shelf (WSS), and warm and salty Atlantic Water (AW), of the West Spitsbergen Current (WSC) along the shelf slope (Fig. 1a) [11]. These water masses mix to varying extent as they are advected into fjords on Spitsbergen, such as Kongsfjorden [12, 13].

Recent studies have shown that the Arctic Ocean is home to microbial communities vulnerable to climate-induced changes, leading to alterations in the productivity and stability of Arctic marine ecosystems [15, 16]. Warming of the Arctic is affecting the distribution of microbial communities, with studies pointing to a shift towards species that thrive in warmer waters, outcompeting native psychrophilic microbes and those depending on ice-algae interactions [16–18]. Polar and Atlantic Waters in the Svalbard region and Fram Strait have distinct prokaryotic and eukaryotic signatures [19–21]. Although structural shifts of prokaryotic communities are foreseen due to Atlantification, also known as borealization (e.g. [10]), they are difficult to predict due to the complexity of factors that affect these community changes [22]. Despite the numerous studies done in some of the Svalbard fjords, with emphasis on Kongsfjorden, the consequences of Atlantification on the prokaryotic communities and biogeochemical cycles are yet to be established.

In this study we contrast the hydrography, biogeochemistry and prokaryotic diversity of the “warm” Kongsfjorden and the “cold” Rijpfjorden (Svalbard). Considering that variations in water masses drive shifts in prokaryotic communities (e.g. [23]), we hypothesize that such shifts can provide insights into the impacts of Atlantification as it progresses eastward along the northern shelf of Svalbard. Characterizing the prokaryotic community of Kongsfjorden, dominated by Atlantic Water (AW), and that of Rijpfjorden, dominated by colder water masses, helps predict major shifts in species composition and diversity if AW expands into this cold fjord. The contrast is not meant to show that fjords differ, but that these differences serve as a natural proxy for future Atlantification scenarios.

We used physical, chemical and microbiological data (16S rRNA gene amplicon and shotgun metagenomic sequencing) collected along two transects in the summer of 2016 and 2017 from the inner parts of Kongsfjorden and Rijpfjorden, across the shelves and into Fram Strait and Nansen Basin, respectively. We complemented our study with physical oceanography data collected in both fjords in 2012, 2013 and 2014.

## Methods

### Study sites

The study includes two fjords in the Svalbard archipelago; their adjacent shelf areas and the deep ocean located in front of them: Kongsfjorden in Spitsbergen (~ 79ºN and 11–13ºE) and Rijpfjorden in Nordaustlandet (~ 80ºN and 21–23ºE) (Fig. 1). The former is one of the best-studied high-latitude fjord systems [24, 25]. Due to its location at the interface of Arctic and relatively warm Atlantic water masses, Kongsfjorden experiences large amplitudes in physico-chemical conditions and might be considered as an early-warning indicator of future changes in Arctic fjords [26]. In the last decades, the heat content of Kongsfjorden has increased, whereas the winter/spring sea-ice extent has decreased [27–29]. Rijpfjorden is a cold fjord influenced by Arctic Water (ArW) for most of the year (January–July) and covered by sea ice for 6–8 months, which often is still present in July/August [30]. Kongsfjorden is 26 km long while Rijpfjorden is approximately 40 km long, but sampling transects extended past the fjords to the shelves and slopes to the deep ocean (Fig. 1).

### Sample collection

Samples were collected in 25–28 July 2016 and 29 July-1 August 2017 in Kongsfjorden, and 29 July-1 August 2016 and 3-5 August 2017 in Rijpfjorden (Fig. 1), on board RV *Lance* as part of the Environmental Monitoring of Svalbard and Jan Mayen (MOSJ) (https://www.mosj.no/en/about/, accessed 14.02.2026). We complemented our study with physical oceanography data collected in both fjords in the summers of 2012, 2013 and 2014 [31]. The year 2015 was not included due to the absence of data for Rijpfjorden.

In the Kongsfjorden transect (Fig. 1c, d), seawater samples were collected at ten stations for physical and biogeochemical measurements (Kb5, Kb3, Kb2, Kb1, Kb0, V12, V10, V6, KH, HG-IV). Six stations (Kb6, Kb3, Kb0, V12, V6 and HGIV) were used for collecting samples for V4-V5 16S rRNA gene amplicon sequencing and for shotgun metagenomic sequencing. In the Rijpfjorden transect (Fig. 1b), seawater samples were collected at nine stations for physical and biogeochemical measurements (R1, R2, R3, R4, R5, R6, R6-b, R7, R7-b, R8 and R9), of which seven (R1, R3, R4, R5, R6, R7 and R8) were sampled for V4-V5 16S rRNA gene amplicon sequencing, and two (R1 and R7) for shotgun metagenomic sequencing. Because of logistical limitations, not all stations were sampled in both 2016 and 2017 for amplicon and metagenomic sequencing (Table S1). In Rijpfjorden, only station R1 was sampled in both years for amplicon sequencing, and no stations were sampled for metagenomic analysis in 2017, which constrains year-to-year comparisons for this fjord.

Water samples were collected with Niskin bottles mounted on a rosette sampler equipped with a CTD (conductivity-temperature-depth, Sea-Bird Electronics, Bellevue, WA, USA), photosynthetically active radiation (400–700 nm, PAR; Spherical underwater Quantum Sensor Li-193, LI-COR Biosciences) and chlorophyll *a* (Chl *a*) fluorescence (WETStar, Sea-Bird Electronics) sensors. Physical data from the CTD sensors was collected with a vertical resolution of 1 m, from the surface to the bottom. Chl *a* was sampled at the surface, 5, 10, 25, 50, 100 m, and at the Chl *a* maximum depth when it differed (> 5 m) from the standard depths. Nutrients (ammonium, nitrate, nitrite, phosphate and silicic acid) were sampled at the same depths and at 200, 400, 600, 1000 m and bottom, depending on total depth. Occasionally, samples were collected from depths differing from those listed above (Table S1).

Samples for V4-V5 16S rRNA gene amplicon and shotgun metagenomic sequencing were collected at three different depths: surface (1–5 m), Chl *a* maximum (5–43 m) and near the bottom (50–1035 m). Exceptions were Kb0 with four depths (5, 25, 50 and 320 m) in 2016 and only one depth (50 m) in 2017, and stations R6 and R8 with two depths (5 and 50 m) in 2016 and five depths (5, 15, 50, 100 and 890 m) in 2017 (Table S1).

### Sample processing for nutrients and microbial community analysis

Subsamples for nitrate, nitrite, phosphate and silicic acid were collected in 20 mL acid-washed scintillation vials, fixed with 0.2 mL chloroform and stored at 4ºC until processing [32]. Their concentrations were determined spectrophotometrically with a modified Skalar autoanalyzer (Skalar Analytical Instruments, Breda, Netherlands) at wavelengths of 540, 540, 810, and 810 nm, respectively. The detection limits were: 0.5 mmol m^−3^ for nitrate, 0.06 mmol m^−3^ for nitrite and phosphate, and 0.7 mmol m^−3^ for silicic acid.

Subsamples for ammonium were collected into 10 mL Falcon polypropylene tubes and analysed immediately onboard. Concentrations were measured spectrophotometrically in triplicates as described by [33], with color development at 50 ºC in a water bath over 1 h. For ammonium blanks, Milli-Q water was used. The detection limit was 0.09 μM.

Water samples were filtered through a 25 mm diameter Whatman GF/F filter for Chl *a* analysis. Chl *a* was extracted in 5 mL 100% methanol for 12 h at 5 ºC in darkness and fluorescence was measured with a Turner Designs AU10 fluorometer (Turner Designs, California, USA) at room temperature. The CTD fluorometer was calibrated using a Chl *a* standard from *Anacystis nidulans* algae (Sigma Aldrich C6144; [34]).

For V4-V5 16S rRNA gene amplicon and shotgun metagenomic sequencing samples, 2 to 4 L of the collected water were filtered through 0.22 µm SterivexTM filters (Millipore) and stored on board in −80ºC until further total DNA isolation. Previous standardization of seawater sampling methodologies for microbiome analysis ensures that samples are comparable for the range of volumes used and for the filters selected [35, 36].

### DNA extraction and sequencing

The Sterivex filters were defrosted at room temperature, and total DNA was extracted using the PowerWater® Sterivex DNA Isolation Kit (Qiagen, Germany) according to the manufacturer’s instructions. This DNA was subsequently used for both 16S rRNA metabarcoding and untargeted metagenomic sequencing. The V4-V5 region of the 16S rRNA gene was amplified using the primer pair 515YF (5´- GTGYCAGCMGCCGCGGTAA—3´) and 926R (5´ CCGYCAATTYMTTTRAGTTT—3´) [37, 38]. Amplicons were prepared for Illumina sequencing (Illumina MiSeq) at LGC Genomics (LGC Genomics GmbH, Berlin, Germany), following the conditions described in detail by Semedo et al. [39].

Total DNA isolated from 15 samples collected in 2016 and 11 samples collected in 2017 from stations in Kongsfjorden and Rijpfjorden was also analysed using shotgun metagenomic sequencing (Table S1). DNA was sheared to about 500 bp fragments using a Covaris S220 sonicator, purified and concentrated by a clean-up using MinElute columns (Qiagen). DNA concentrations were measured and 100 ng were used to prepare Illumina libraries. Libraries were done with the Ovation Rapid DR multiplex 1–96 system (NuGEN) and amplified using standard Illumina primers for 8 to 15 cycles with MyTaq (Bioline). Sequencing was done on an Illumina NextSeq 500. All procedures outlined above were carried out by LGC Genomics (Berlin, Germany).

### Bioinformatic processing of sequencing results

Bioinformatic processing of raw reads from V4-V5 16S rRNA gene sequencing into Amplicon Sequence Variants (ASVs) was conducted as described in detail previously [39–41]. Primers from the raw FastQ files obtained from Illumina MiSeq sequencing were removed using “cutadapt v.1.16”. Files were imported into R (v 4.1.1) (R Core Team, 2023) and processed with the DADA2 R package (v 1.20.0) [42]. Forward and reverse reads trimming parameters were decided based on a compromise between the read size of both 2016 and 2017 data (Forward = 249 nt, Reverse = 214 nt; [41]), the pool argument was set to FALSE, *i.e.,* ASV were estimated per sample. The remaining parameters were set to default values [42]. Taxonomy was assigned using the naive Bayesian classifier [43] with the GTDB v202 reference database [44, 45], using the latest available pre-trained set for taxonomic classification by DADA2 that was available at time of analysis. Taxonomic nomenclature was used as provided by the database. Therefore, some groups do not reflect the latest nomenclature updates, which is the case for Proteobacteria (now Pseudomonadota). The summary of reads after each processing step is available in Supplementary Table S2.

To survey taxonomic groups implicated in nitrification, we selected ammonium-oxidizing archaea (AOA), ammonium-oxidizing bacteria (AOB) and nitrite-oxidizing bacteria (NOB). For AOA we surveyed ASVs from the class Nitrososphaeria; for AOB we surveyed ASVs from the family Nitrosomonadaceae or from the genus *Nitrosococcus*; and for NOB we surveyed ASVs from the phyla Nitrospinota and Nitrospirota and from the genera *Nitrotoga* and *Nitrobacter* [39, 46].

The raw shotgun metagenomic reads were trimmed with Trimmomatic v0.36, to remove adapter sequences, short reads (< 36 bp), and reads with an average quality score < 15 within 4-base windows [47] (Table S3). De novo assembly of the reads was performed using metaSPAdes v3.15.3 [48, 49], with a minimum contig length of 2000 bp. Functional annotation of genes of the assembled contigs was done using PROKKA v1.14.5 [50]. Gene abundance was estimated by mapping the trimmed paired reads back onto the contigs using bowtie2 [51], with local alignment mode and allowing 1 bp mismatch. The targeted genes of this study were the ones implicated in the nitrogen cycle (Table S4), based on previous literature [39, 52]. The number of reads mapped to the target genes was counted in the metagenomic samples, using SAMtools [53]. As a measure of gene abundance, we quantified the coverage [54] of each of the nitrogen cycle target genes found in the metagenomes. This method involved counting the number of reads mapping to each of the contigs divided by the length (in bp) of the gene [55]. To take into account the differences of sequencing depth between samples, the gene coverage was then normalized against the mean coverage of three reference single-copy genes: RecA protein (*recA*), DNA gyrase subunit B (*gyrB*), and DNA-directed RNA polymerase subunit beta (*rpoB*), and expressed as “Average Genomic Copy Number”, according to what has been described in detail by Semedo et al. [55].

### Water mass classification

We used the water mass definitions described in [13]: Arctic Water (ArW), Atlantic Water (AW), Intermediate Water (IW), Local Water (LW), Surface Water (SW), Transformed Atlantic Water (TAW) and Winter-cooled Water (WCW), following the envelopes specified in Table S5. According to Cottier et al. [13], σ threshold (< 27.92 kg m^−3^) should be used in the envelope of AW and TAW. However, we found some cases falling within the temperature and salinity limits for either AW or TAW and with a σ above the mentioned threshold that did not fall within any of the other envelopes. In these cases, we assumed this to be AW or TAW, depending only on temperature and salinity limits. We also found that the partial overlap of the envelopes of ArW, LW and WCW made their distinction difficult. In some cases, we found water masses with less salinity than the lower limit of ArW and with a temperature lower than the lower limit of LW. We classified them as ArW. We focused the comparative analysis between Kongsfjorden and Rijpfjorden and adjacent shelves on these water masses. This was to distinguish differences due to the relative fractions of the water masses from differences in the biogeochemical properties of each water mass (e.g., [56]).

### Statistical analysis

Water masses as well as physical and biogeochemical variables are presented in vertical transect plots for each fjord. Some of these plots contain gaps due to the lack of data for given stations/depths.

Our physical and biogeochemical data consist of measurements collected at multiple depths per station, implying vertical autocorrelation and potentially skewed distributions that violate the assumptions of parametric statistical tests. Therefore, station data were aggregated per year (2016 and 2017) and per water mass by calculating station-level medians, which were then used as independent observations in the statistical analyses. This aggregation approach minimizes dependence among observations while preserving station-level variability. Differences in the concentrations of ammonium, nitrate + nitrite, phosphate, silicic acid, and Chl *a* between the two fjords were evaluated for comparable water masses using the Mann–Whitney U test [57]. For descriptive purposes, we computed overall medians, 25th and 75th percentiles, arithmetic means, and 95% bootstrap confidence intervals for each water mass [58], from the aggregated medians.

Stoichiometric ratios between dissolved inorganic nitrogen (DIN = nitrate + nitrite + ammonium) and phosphorus, as well as between DIN and silica, were calculated for all water masses. Mixing diagrams were constructed for nutrients [59–61] and for the different water masses. Associations between nutrient concentrations and salinity were summarized using standardized major axis and Deming (Model II) regression (e.g. [62] and [63]). Because Deming regression with λ = 1 (assuming both variables are equally uncertain) is equivalent to major axis regression, it was used solely to obtain uncertainty estimates and significance tests for the major axis slope.

Since multiple statistical tests were performed, confidence levels were adjusted using the Benjamini–Hochberg (FDR) false discovery rate correction [64, 65].

We used salinity and nutrient concentration changes between the shelf and the fjords, in different water masses to compute biogeochemical sink/sources following the method described in [66], based on the usage of Eq. 1.

1 $$\begin{aligned} &Sources-Sinks={C}_{fjord}-{C}_{cons}\\&={C}_{fjord}-[{C}_{0}+\left({S}_{fjord}-{S}_{0}\right).\left(\frac{{C}_{shelf}-{C}_{0}}{{S}_{shelf}-{S}_{0}}\right)] \end{aligned}$$

Where *S* and *C* represent salinity and nutrient concentration. The subscript “*o*” stands for freshwater sources. The subscripts “fjord” and “shelf” indicate the regions used to compute average salinities and concentrations in selected water masses. *C*_*cons*_ is calculated with the term in square brackets.

The following shelf stations were considered for Kongsfjorden and Rijpfjorden, respectively: V10, V12, V14, and R4, R5, R6, R6-b, R7 and R7-b. The following fjord stations were considered for Kongsfjorden and Rijpfjorden, respectively: Kb0-Kb5 and R1-R3. The same water masses were used to compute differences between values in the shelf and values in the fjord, in both years, in the case of Kongsfjorden: AWs (Atlantic Water in the shelf) versus IW, SW or AWf (Atlantic Water in the fjord). In the case of Rijpfjorden, different water masses were used in 2016 and in 2017 due to major differences in the hydrographic conditions. Therefore, in 2016 we computed differences in salinity and concentration of nitrate + nitrite for TAWs (Transformed Atlantic Water in the shelf) versus IW, SW or TAWf (Transformed Atlantic Water in the fjord), whereas in the 2017 we computed differences for ArWs (Arctic Water in the shelf) versus ArWf (Arctic Water in the fjord). In all cases, when water masses are indicated without “s” or “f” to specify shelf and fjord values, they refer to fjord values only. Calculations were restricted to the top 100 m and assumed zero salinities and nutrient concentrations for freshwater endmembers. The no-nutrient assumption was to make calculations comparable for both transects, since to the best of our knowledge there are no nutrient concentration data for Rijpfjorden freshwater sources. This assumption makes it more difficult to identify biogeochemical sinks/sources since we positively bias the dilution effect of freshwater.

Microbiome statistical analysis was performed in R [67]. We verified that the sequencing effort was enough by plotting rarefaction curves for V4-V5 16S rRNA gene amplicon (Fig. S1). To estimate alpha diversity, we used the number of taxa in a given sample (taxa richness). Beta diversity was accessed by calculating Bray–Curtis distance of relative abundance scores from taxa between samples, using non-Metric Multidimensional Scaling (nMDS) for dimensionality reduction and ordination, with the metaMDS function from Vegan R package [68]. Significance was accessed with Permutational MANOVA (PERMANOVA) using the adonis2 function; and homogeneity of group dispersion was evaluated with the betadisper function of the same R package.

For the subset of nitrifiers in the 16S rRNA gene amplicon sequencing, alpha and beta diversities were calculated using the same functions that were applied to the entire community. No rescaling of relative abundance to 100% was applied to the nitrifier subset.

## Results

### Hydrography

Our focus is on the summers of 2016 and 2017 for which we have both hydrographic and microbiological data. However, we include hydrographic data for 2012–2014 in the Supporting Information file to identify the dominant hydrography patterns of both fjords.

**Fig. 2:**
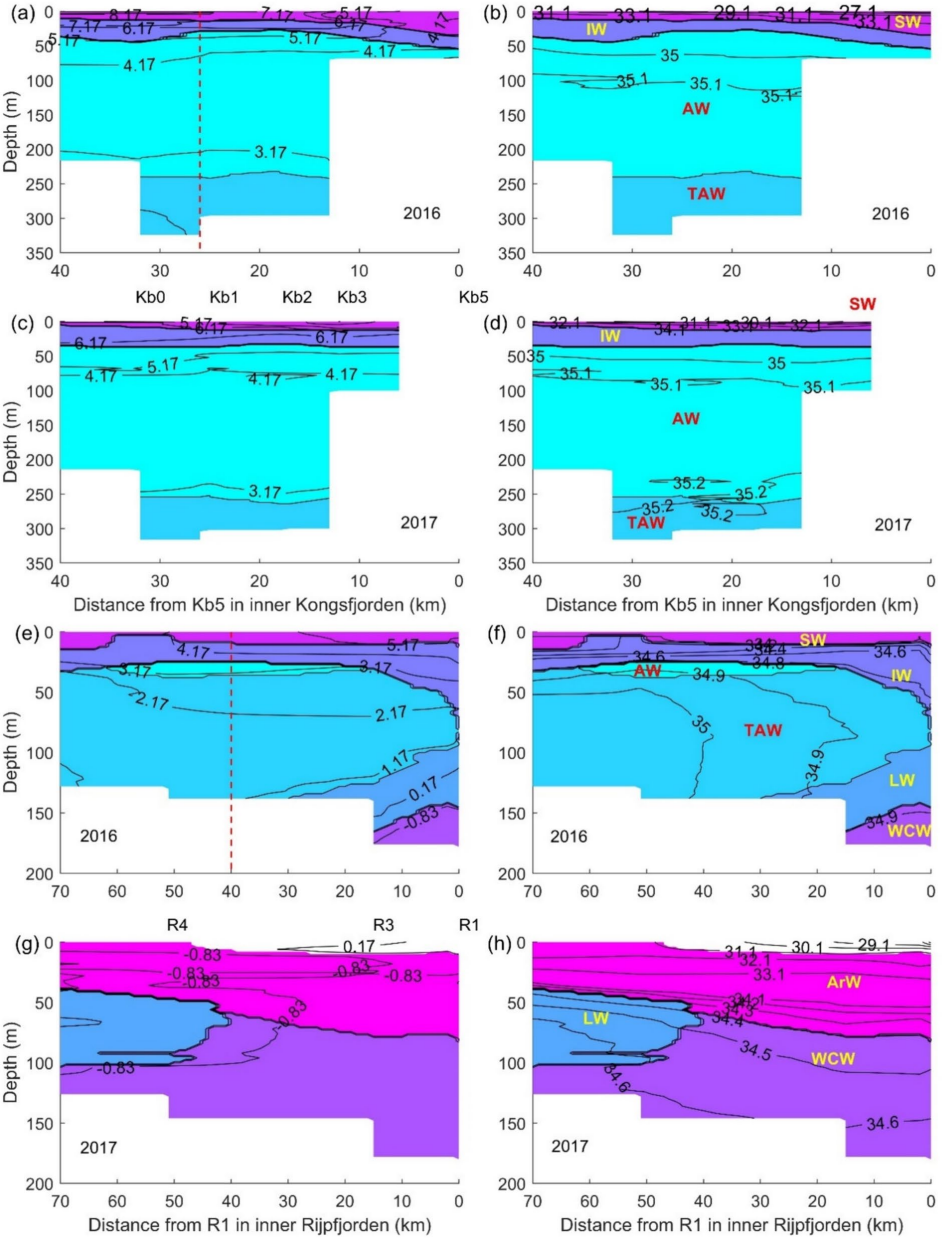
Isotherms (conservative temperature in °C, left panels), isohalines (absolute salinity, right panels) and water masses (colour scale) in Kongsfjorden, in 2016 and 2017 (**a**, **b** and **c**, **d**, respectively), and Rijpfjorden in 2016 and 2017 (**e**, **f** and **g**, **h**, respectively). The correspondence between color scale and water masses is shown in panels (**b**), (**d**), (**f**) and (**h**): Arctic Water (ArW), Atlantic Water (AW), Intermediate Water (IW), Local Water (LW), Surface Water (SW), Transformed Atlantic Water (TAW) and Winter-cooled Water (WCW). Water masses are identified following the temperature-salinity envelopes described in Cottier et al. [13]. The vertical red dashed lines in panels (**a**) and (**e**) mark the mouth of the fjords. Results are shown only for the fjords and adjacent shelves. The location of some of the sampling stations depicted in Fig. 1 is also shown in panels **c** and **g**

Transformed Atlantic Water (TAW) was the dominant water type in 2012 and 2014, inside Kongsfjorden, whereas the warmer Atlantic Water (AW) dominated in 2013, 2016 and 2017 (Fig. S2a, c, e, g, i and S3a, c, e, g, i). The cold Arctic Water (ArW), Local Water (LW) and Winter Cooled Water (WCW) water masses dominated in Rijpfjorden in all years except in 2016 when TAW was the dominant water mass (Fig. S2b, d, f, h, j and S3b, d, f, h, j). Thus, the summers of 2016 and 2017, which are the focus of our study, show similar hydrography in Kongsfjorden but contrasting conditions in Rijpfjorden, where 2016 was a particularly “warm” year (Fig. 2).

The predominance of AW in Kongsfjorden extended offshore, followed at depth (> 300 m) by a layer of TAW. Winter Cooled Water was present off the shelf at > 900 m depth (Fig. 2a, b, and Fig. S4a, c and S5a, c, for details of the deep basin). Surface Water followed by IW was found on the top ~ 50 m, between stations Kb5 and V12. The thickness of these two layers increased towards the inner fjord, whereas AW decreased.

The top layers of SW and IW extended from the inner fjords, along the shelf and into the deep Nansen Basin, in 2016, over a much larger distance in Rijpfjorden than in Kongsfjorden (> 160 km versus ~ 60 km). In Rijpfjorden, AW was found in 2016 in the Nansen Basin (between ~ 100 and 300 m) and on the shelf, as a narrow layer at ~ 25 m depth (Fig. S4b, S5b, 2e, f). In 2016, TAW dominated over most of the surveyed area, except for the inner part of Rijpfjorden (stations R1 and R2), where IW reached its largest thickness and LW as well as WCW were also found. In the Nansen Basin, a layer of WCW was found between ~ 1000 m depth and the bottom. In 2017, the fjord was dominated by cold water masses, as mentioned above. A layer of ArW extended over the whole transect area between the surface and ~ 50 m depth, followed by WCW, in the fjord, LW or TAW on the shelf and Nansen Basin. A layer of AW was present in the Nansen Basin between ~ 100 and 400 m depth (Fig. 2g, h and Figs. S4d and S5d).

Temperatures of SW on the Kongsfjorden shelf reached values > 5 °C in both years, whereas in Rijpfjorden, comparable values were observed only in 2016 (Fig. 2). These differences plus the larger fraction of AW in Kongsfjorden, showed that the heat content in Kongsfjorden and in its adjacent shelf was higher than that in Rijpfjorden over similar depth ranges and in both years. The coldest water masses (ArW, LW and WCW) were not found inside Kongsfjorden but occurred in Rijpfjorden. In both fjords, the lowest salinities (~ 30) were observed close to the glacial fronts but only over a very small area (Fig. 2b, d, f, h).

In both fjords, the isopycnic lines showed stronger gradients towards the surface (Figs. S6 and S7), in the transitions between the AW and the surface layers of IW and SW, in Kongsfjorden, and between TAW and IW, in Rijpfjorden in 2016. In Rijpfjorden in 2017, the larger density differences were between LW or WCW versus ArW.

### Nutrients and Chl *a*

**Fig. 3:**
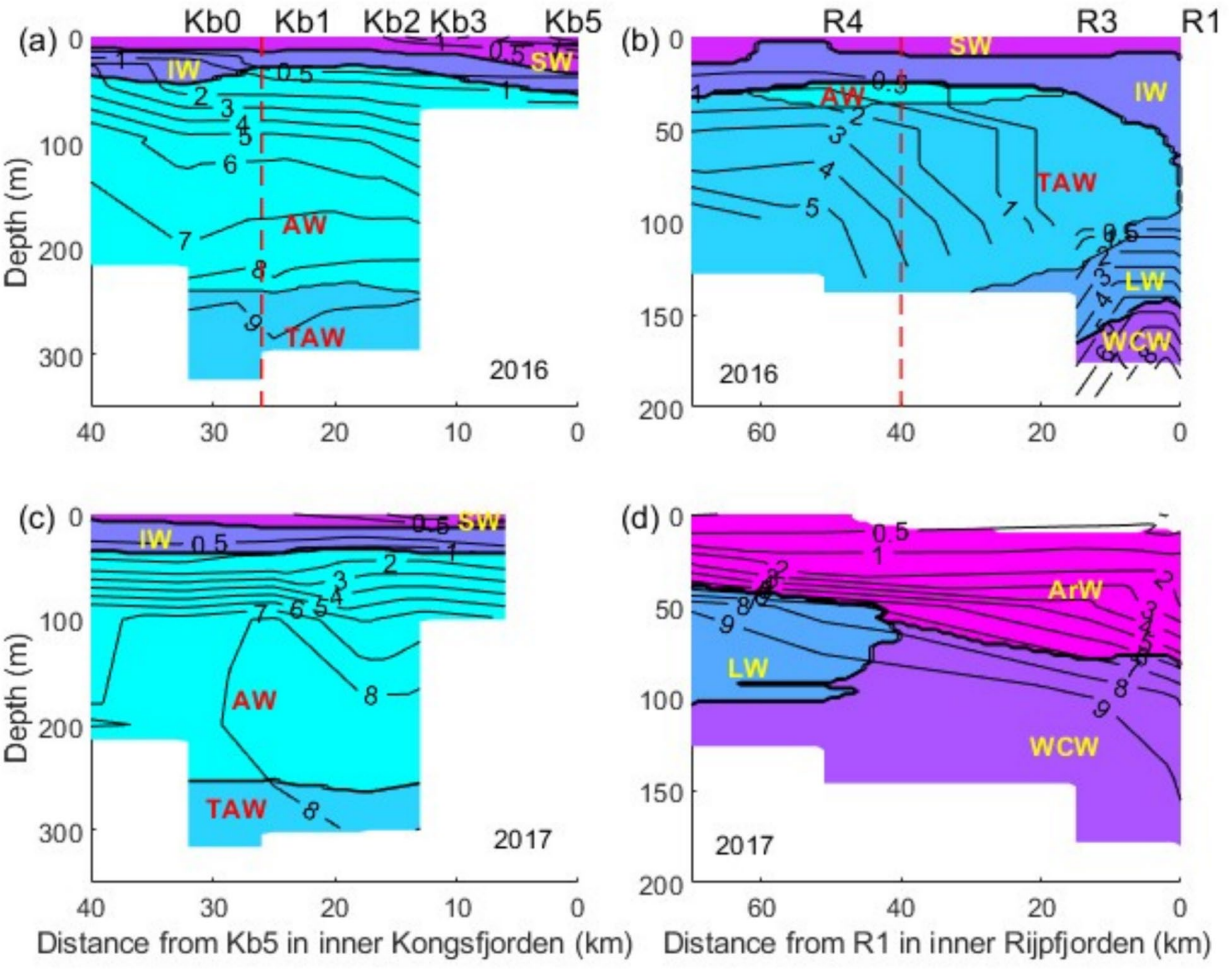
Nitrate + nitrite concentration isolines (mmol m^−3^) and water masses (labels as in Fig. 2) for 2016 (top panels) and 2017 (bottom panels). **a** and **c** Kongsfjorden transect. **b** and **d** Rijpfjorden transect. Results are shown only for the fjords and adjacent shelves. The vertical red dashed lines mark the mouth of the fjords. See Fig. 1 and methods, sample collection

Median and average concentrations of nutrients and Chl *a*, along with their 95% confidence limits are presented per water mass for the fjords (Table 1) and for the fjords and shelves (Table S6). Nitrate + nitrite concentrations were > 8 mmol m^−3^ in the deeper water masses (> 200 m) and < 1.0 mmol m^−3^ in SW and IW (Kongsfjorden 2016 and 2017 and Rijpfjorden 2016) or ArW (Rijpfjorden 2017) (Fig. 3 and Fig. S8). The nitracline was in AW and TAW or within the ArW layer in Rijpfjorden in 2017. Isolines were tilted within the top ~ 150 m, with the thickness of the low nitrate + nitrite surface layer increasing from the shelf into the fjords. Ammonium concentrations were minimal in the deeper layers at the shelf break outside Kongsfjorden (station V6), and Rijpfjorden (station R6) with values of ~ 0.2–0.3 mmol m^−3^. From station V12 into inner Kongsfjorden, values increased towards the surface, up to 2–3 mmol m^−3^ (Fig. S9). In Rijpfjorden, such an increase was not observed in 2016 and ammonium concentration decreased in the inner fjord (Fig. S10). However, in 2017 a slight increase in ammonium towards the inner Rijpfjorden was also observed. Phosphate concentrations in both fjords were < 1 mmol m^−3^, decreasing towards the inner stations and increasing slightly with depth in the shelves and deep ocean (Figs. S11 and S12). Silicic acid concentrations had a range between > 10 mmol m^−3^ in WCW and ~ 1–2 mmol m^−3^ in SW and IW or ArW (in the case of Rijpfjorden 2017) (Figs. S13 and S14). Chl *a* concentrations were generally < 1.0 mg m^−3^ close to the surface and then decreasing with depth and reaching values ~ 0.1–0.2 mg m^−3^ at ~ 100 m depth in Kongsfjorden and ~ 0.4 in Rijpfjorden (Fig. S15).

**Table 1:** Descriptive statistics for nutrients and Chl *a* concentrations, in different water masses [Atlantic Water (AW), Arctic Water (ArW), Intermediate Water (IW), Local Water (LW), Surface Water (SW), Transformed Atlantic Water (TAW) and Winter-cooled Water (WCW)], from fjord stations Kb1—5 and R1-3 in Kongsfjorden (K) and Rijpfjorden (R), respectively, using data from summers 2016 and 2017. A dash is used when data is absent. Bold type indicates significant differences (*p* < 0.05) between fjords with the Mann–Whitney U test. We show the probability of Type I error returned from each test (*p*) and corrected (*p_corr*) with the Benjamini–Hochberg false discovery rate correction for multiple testing [65]. Confidence limits (Low and High CL) were computed with the bootstrap method (cf.—Methods, Statistical analysis). Refer Fig. 1 for the location of the sampling stations and Table S5 for the water mass classification

Variable	Water mass	Sample size		Medians		Means	Low CL	High CL	Means	Low CL	High CL	Mann–Whitney	
		**K**	**R**	**K**	**R**		**K**			**R**		***p***	***p_corr***
Ammonium	AW	9	0	1.78	-	1.87	1.59	2.30	-	-	-	-	-
	ArW	0	3	-	0.64	-	-	-	0.64	0.54	0.75	-	-
	IW	9	3	0.87	0.18	1.16	0.86	1.66	0.17	0.00	0.32	**0.01**	**0.03**
	LW	0	5	-	0.92	-	-	-	0.97	0.59	1.34	-	-
	SW	9	6	0.46	0.25	0.76	0.40	1.23	0.26	0.12	0.42	**0.05**	0.10
	TAW	8	1	1.38	1.42	1.32	0.83	1.83	1.42	-	-	-	-
	WCW	0	6	-	0.77	-	-	-	1.59	0.52	2.97	-	-
Nitrate + nitrite	AW	9	0	5.69	-	5.66	4.58	6.68	-	-	-	-	-
	ArW	0	3	-	1.21	-	-	-	1.20	0.85	1.56	-	-
	IW	9	3	0.47	0.00	0.47	0.22	0.75	0.00	0.00	0.00	**0.01**	**0.03**
	LW	0	5	-	2.40	-	-	-	2.56	0.48	4.63	-	-
	SW	9	6	0.26	0.16	0.25	0.13	0.39	0.25	0.05	0.48	0.67	0.83
	TAW	8	1	9.07	0.00	9.13	8.12	10.19	0.00	-	-	-	-
	WCW	0	6	-	9.24	-	-	-	8.87	8.02	9.65	-	-
Phosphate	AW	9	0	0.59	-	0.54	0.46	0.60	-	-	-	-	-
	ArW	0	3	-	0.15	-	-	-	0.17	0.15	0.20	-	-
	IW	9	3	0.17	0.12	0.16	0.14	0.17	0.12	0.11	0.13	**0.04**	0.09
	LW	0	5	-	0.32	-	-	-	0.30	0.16	0.44	-	-
	SW	9	6	0.11	0.09	0.11	0.09	0.13	0.10	0.07	0.15	0.28	0.40
	TAW	8	1	0.78	0.21	0.78	0.72	0.84	0.21	-	-	-	-
	WCW	0	6	-	0.73	-	-	-	0.75	0.66	0.83	-	-
Silicic acid	AW	9	0	2.93	-	2.91	2.47	3.27	-	-	-	-	-
	ArW	0	3	-	1.72	-	-	-	1.79	1.58	2.08	-	-
	IW	9	3	0.99	1.69	1.00	0.86	1.15	1.69	1.57	1.80	**0.01**	**0.03**
	LW	0	5	-	2.84	-	-	-	1.99	0.57	3.41	-	-
	SW	9	6	1.09	1.60	1.19	0.93	1.47	1.52	1.24	1.78	0.11	0.19
	TAW	8	1	5.91	1.60	6.75	5.36	8.85	1.60	-	-	-	-
	WCW	0	6	-	4.62	-	-	-	4.29	3.53	4.87	-	-
Chl *a*	AW	9	0	0.14	-	0.17	0.11	0.23	-	-	-	-	-
	ArW	0	3	-	1.03	-	-	-	1.14	1.01	1.39	-	-
	IW	9	3	0.31	0.33	0.43	0.23	0.65	0.38	0.30	0.51	0.86	0.95
	LW	0	5	-	0.29	-	-	-	0.28	0.10	0.49	-	-
	SW	9	6	1.04	0.79	1.11	0.72	1.58	1.02	0.63	1.46	0.95	0.95
	TAW	0	1	-	0.08	-	-	-	0.08	-	-	-	-
	WCW	0	3	-	0.26	-	-	-	0.31	0.24	0.44	-	-

Except for ammonium, TAW exhibited the highest nutrient concentrations in Kongsfjorden, followed by AW (Table 1, S6, Figs. S16 and S17). In the case of Rijpfjorden, WCW had the highest nutrient concentrations, seconded by TAW, AW and, LW and IW in 2017 (Figs. S16 and S17). Lowest nutrient concentrations occurred in SW and ArW. We compared nutrient and Chl *a* concentrations per water mass between the two fjords, using only fjord stations or fjord and shelf stations (Tables 1 and S6, respectively). In the fjord-only case, we found significant differences only for IW (Mann–Whitney U test, *p* < 0.05) and for all nutrients (Kongsfjorden values being larger than Rijpfjorden values, except for silicic acid). However, when we corrected the significance level with the Benjamini–Hochberg false discovery rate correction for multiple testing [65], we could not confirm the significance of the differences in phosphate between the two fjords. When we included fjord and shelf stations in the comparisons we found significant differences for ammonium (Kongsfjorden > Rijpfjorden) in AW, IW and SW, nitrate + nitrite, in AW (Rijpfjorden > Kongsfjorden), phosphate, in AW (Rijpfjorden > Kongsfjorden) and SW (Kongsfjorden > Rijpfjorden), and silicic acid, in AW, IW (Rijpfjorden > Kongsfjorden) and TAW (Kongsfjorden > Rijpfjorden). However, the significance of these differences was not confirmed after the Benjamini–Hochberg correction, except for ammonium in IW and SW. We found no significant differences for Chl *a.* Nutrient concentration differences between water masses were generally larger than differences between 2016 and 2017 for the same water mass (Figs S16 and S17). In the cold 2017 Rijpfjorden showed less surface nutrient depletion between the shelf and the fjord stations (in the top layer of ArW) than in 2016 (in the top layers of SW and IW).

Dissolved inorganic nitrogen to phosphorus molar ratios were lower than Redfield N: P atom ratio of 16:1 for all water masses and for both study sites, with a few exceptions, especially for SW in Kongsfjorden due to its relatively high ammonium content (Figs. S18a, c, e, S19a, c, S20a, c). Dissolved inorganic nitrogen to silica molar ratios were predominantly > 1 for AW, TAW (Fig. S18d, f), LW and WCW (Fig. S20b, d), and variable for the remaining water masses (Figs. S18b and S19b, d).

We constructed salinity–nutrient mixing diagrams separately for Kongsfjorden and Rijpfjorden for the years 2016 and 2017. Across all nutrients, fjords, years, and water masses, significant standardized major axis/Deming regressions with salinity were obtained in 28 out of 72 mixing diagrams (Figs. S21–S24; Table S7).

Considering only the significant regressions summarized in Table S7, negative slopes were observed in Rijpfjorden for ammonium in LW in 2016 and in WCW in 2017. In Kongsfjorden, negative slopes were found for nitrate + nitrite in TAW in 2016 and in SW in 2017, as well as for silicic acid in SW in both years.

Positive slopes for ammonium were limited to TAW in Kongsfjorden in 2016 and ArW in Rijpfjorden in 2017. For the other nutrients, positive slopes were primarily observed in Kongsfjorden for AW in both years, except for phosphate, which was significant only in 2016. In Rijpfjorden, positive slopes for nitrate – nitrite, phosphate and silicic acid were detected in TAW in 2016 and in ArW, LW, and WCW in 2017. Additional positive relationships were observed for phosphate in IW and AW in 2016 and 2017, respectively, and for silicic acid in WCW in 2016.

We used Eq. 1 to compute nutrient biogeochemical Sources-Sinks (Table 2, S8-S10, for nitrate + nitrite, ammonium, phosphate and silica acid, respectively). We obtained negative values (biogeochemical sink) in all cases (Kongsfjorden and Rijpfjorden, 2016 and 2017) for all nutrients but ammonium, except when comparing AW on the shelf with that in the fjord for Kongsfjorden in 2017. Values for Rijpfjorden 2017 are close to zero. In the case of ammonium, we obtained low positive values (biogeochemical source) in all cases except for Rijpfjorden in 2016. However, the positive values for ammonium are much smaller than the negative values for nitrate + nitrite.

**Table 2:** Biogeochemical nitrate + nitrite *Sources-Sinks* for Kongsfjorden and Rijpfjorden in 2016 and 2017, estimated using Eq. 1, based on differences of average salinities and concentrations of nitrate + nitrite found in the shelf stations in endmember water masses and in fjord stations in “diluted” water masses. See methods, statistical analysis for details and Fig. 1 for the locations of shelf and fjord stations

	Nitrate + nitrite ***Sources-Sinks*** (μmol kg^−1^)					
Kongsfjorden				Rijpfjorden		
Year	AWs ***vs*** IW	AWs ***vs*** SW	AWs ***vs*** AWf	TAWs ***vs***IW	TAWs ***vs*** SW	TAWs ***vs*** TAWf
2016	−5.2	−4.9	−2.5	−6.4	−6.2	−6.4
				ArWs *vs* ArWf −0.5		
2017	−3.3	−3.1	0.5			

### Prokaryotic diversity across transects

**Fig. 4:**
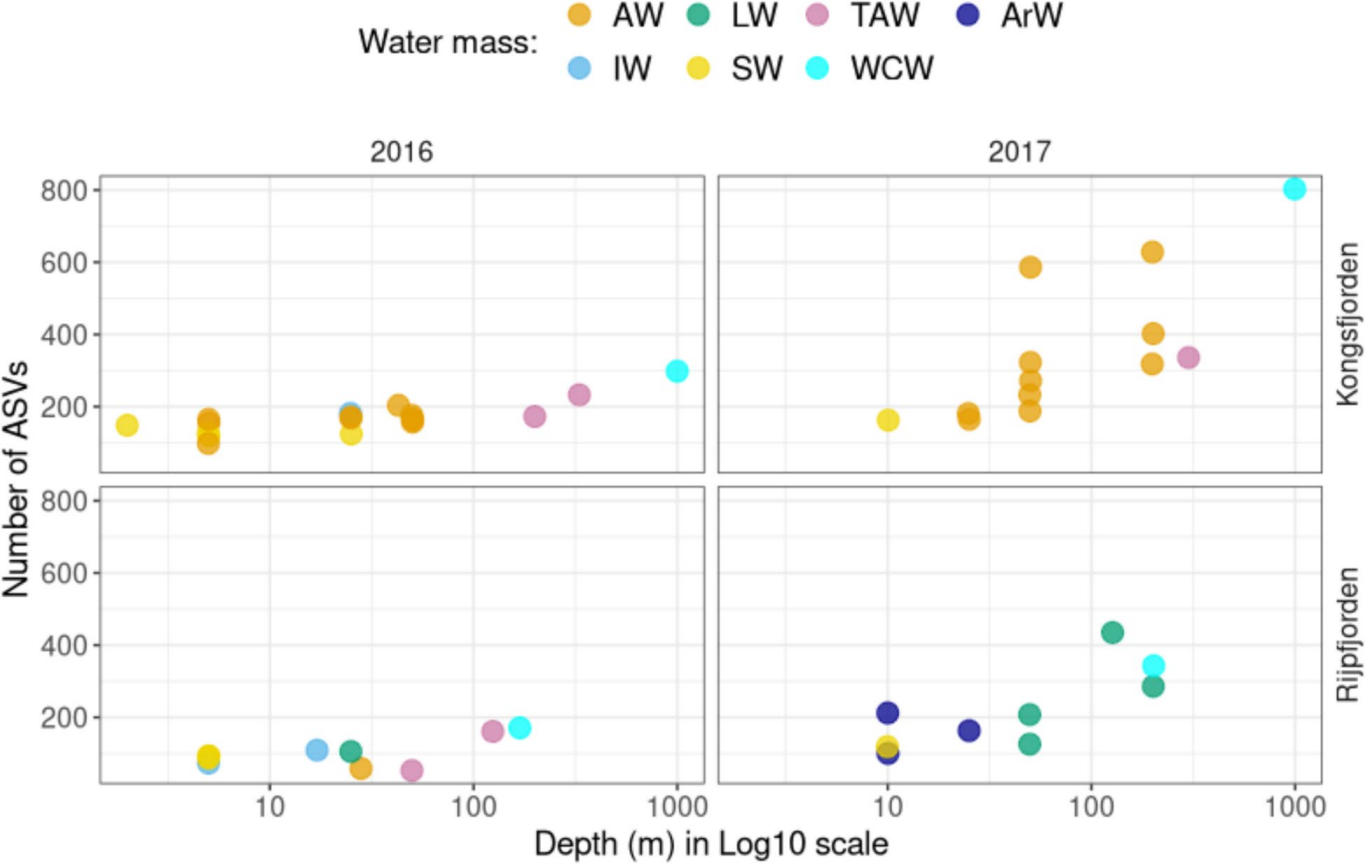
Alpha diversity as a function of depth (in log10 scale), coloured by water mass, for Rijpfjorden and Kongsfjorden transects in 2016 and 2017. Alpha diversity was measured as the number of ASVs in a sample, from V4-V5 16S rRNA gene sequencing data

Details on the number of sequencing readings and their quality are provided in Text S1. Alpha diversity, measured as the number of ASVs per sample, increased with depth below 100 m in both transects and, in both years, with the highest values being at approximately 1000 m, corresponding to the WCW water mass. In 2017, higher alpha diversity was registered in both transects (Fig. 4), and a high variability in ASV richness was observed in the AW of Kongsfjorden, even among samples collected at similar depths.

**Fig. 5:**
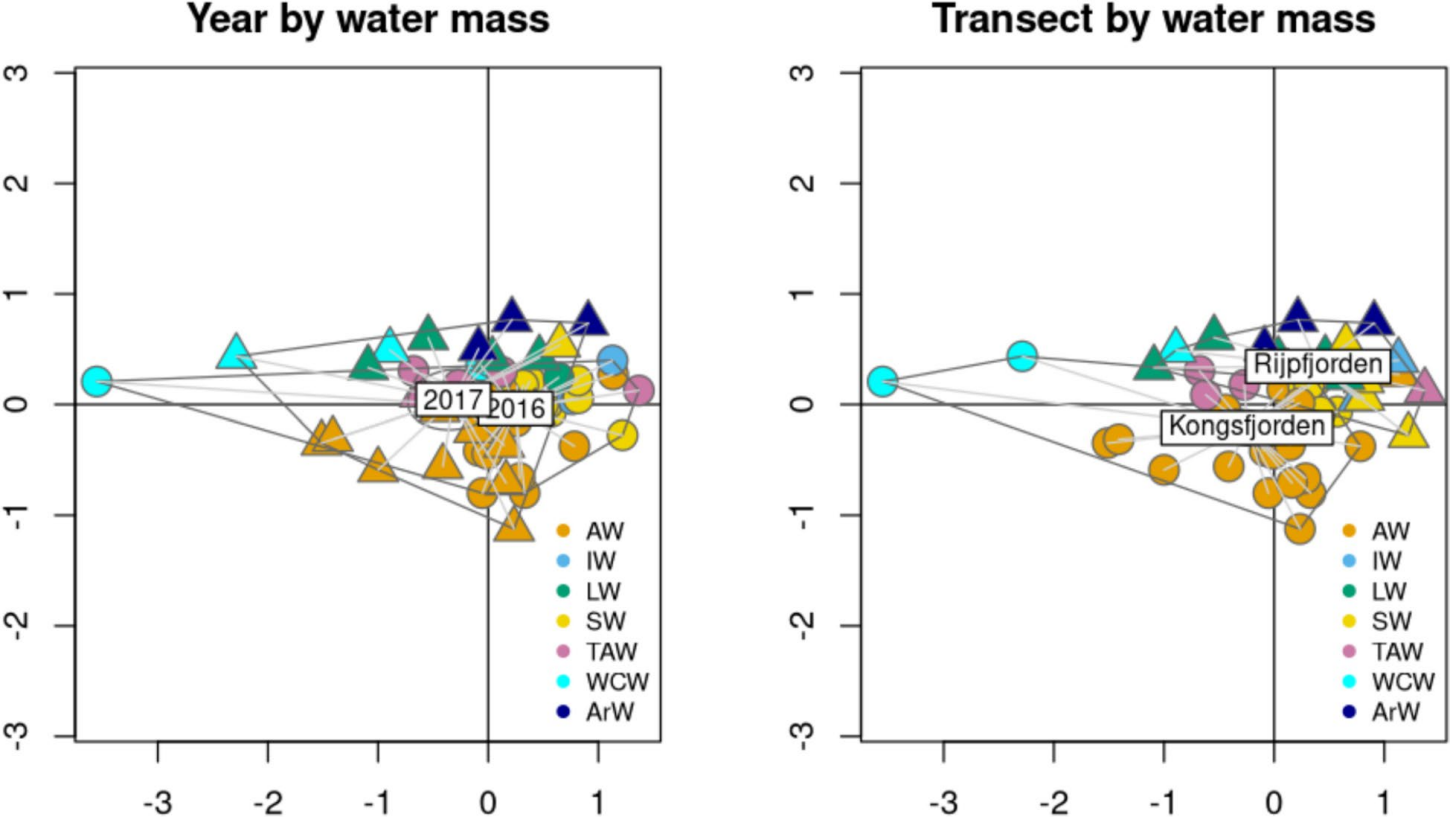
Beta diversity (as nMDS ordination plot), from V4-V5 16S rRNA gene sequencing data, with focus on year using circles for 2016 and triangles for 2017, and transect, using circles for Kongsfjorden and triangles for Rijpfjorden. The label indicates the centroid position of each clustering group, which can be either years or transect. Aspect ratio was set to 1 and stress values were set to 0.128

Looking into the beta diversity, most of the samples from Kongsfjorden and Rijpfjorden were separated along the vertical axis, showing different prokaryotic community composition (Fig. 5). Differences between community composition for the different years were less marked than differences between the two fjords (Fig. 5). In the case of Kongsfjorden, there were many samples from AW dispersed over the bottom quadrants showing large variability in the community composition of this water mass (Fig. 5). Prokaryotic community composition was significantly different between water-masses, depths, years, and transects (*p* < 0.05, PERMANOVA, Table S11). The same test was run to account for the effects of nutrient concentrations, salinity and temperature, showing significant differences for ammonia, nitrate, nitrite, salinity and temperature (*p* < 0.05, PERMANOVA, Table S12) regardless of year (*p* < 0.05, PERMANOVA, Table S13).

**Fig. 6:**
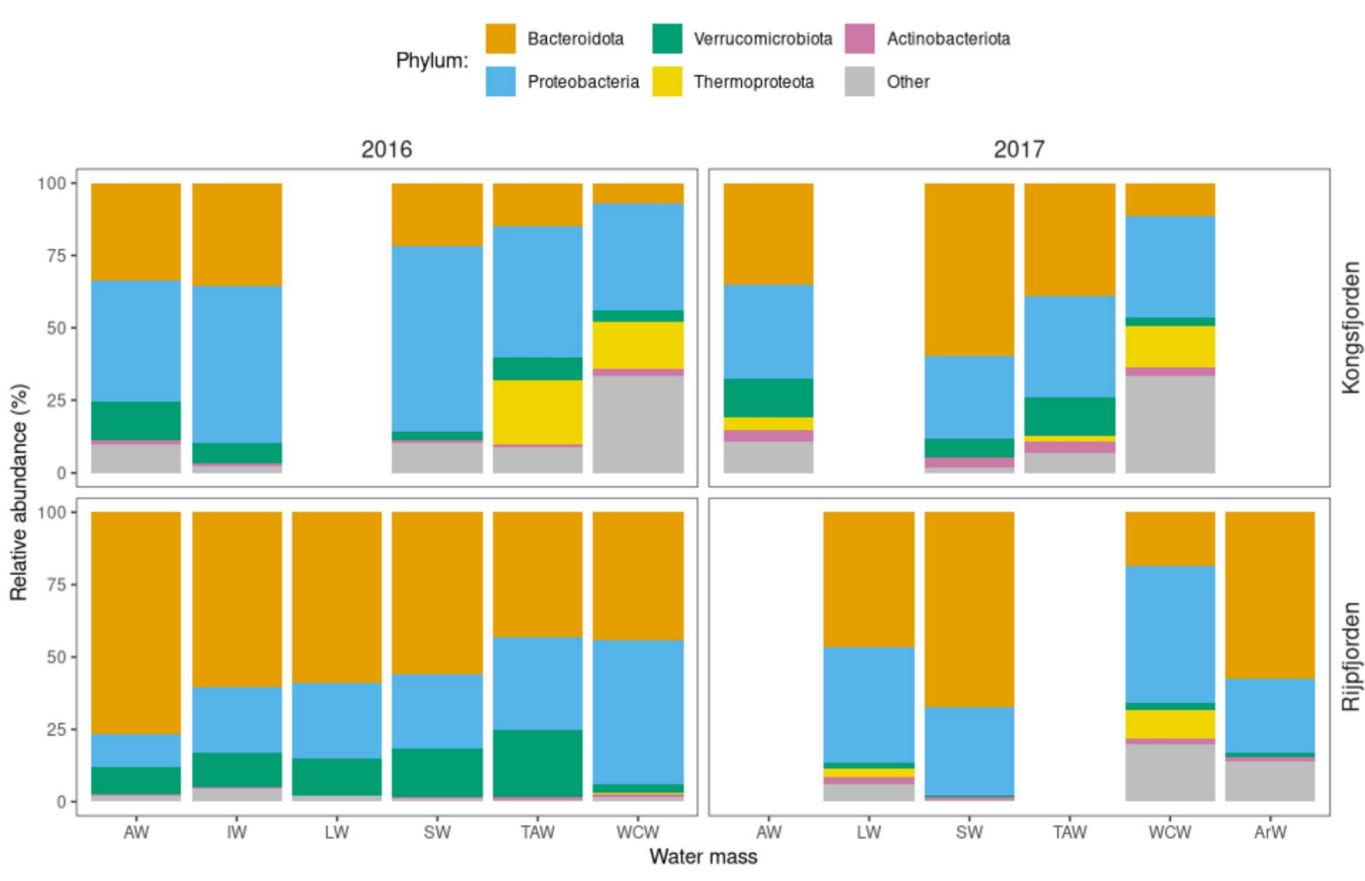
Relative abundance (%) at phylum level, from V4-V5 16S rRNA gene sequencing of samples collected in 2016 and 2017. The top grids correspond to Kongsfjorden and the bottom grids correspond to Rijpfjorden; the left grids correspond to 2016, and the right grids to 2017. The most abundant phyla were selected for qualitative colouring, while the remaining were grouped as “Other”. Blank spaces indicate the absence of a specific water mass

Differences between fjords at the phylum level were evidenced by the higher relative abundance of Proteobacteria in Kongsfjorden and Bacteroidota in Rijpfjorden (Fig. 6), more pronounced in 2016 than in 2017. Differences between sampling years were evident in both fjords. In Kongsfjorden, Thermoproteota was detected only in TAW and WCW in 2016, whereas it was also identified in AW in 2017. The relative abundance of Thermoproteota in Rijpfjorden was low in 2016 and restricted to WCW, whereas it was much higher in 2017, in LW and WCW. Moreover, the relative abundance of Verrucomicrobiota decreased in 2017, in Rijpfjorden.

Within Proteobacteria, genus-level taxonomy revealed that *Pelagibacter* and *Sulfitobacter* had higher relative abundances in Kongsfjorden and Rijpfjorden, respectively (Fig. S25). In addition, *Polaribacter* genus was responsible for the higher relative abundance of *Bacteroidota* in Rijpfjorden (Fig. S26).

**Fig. 7:**
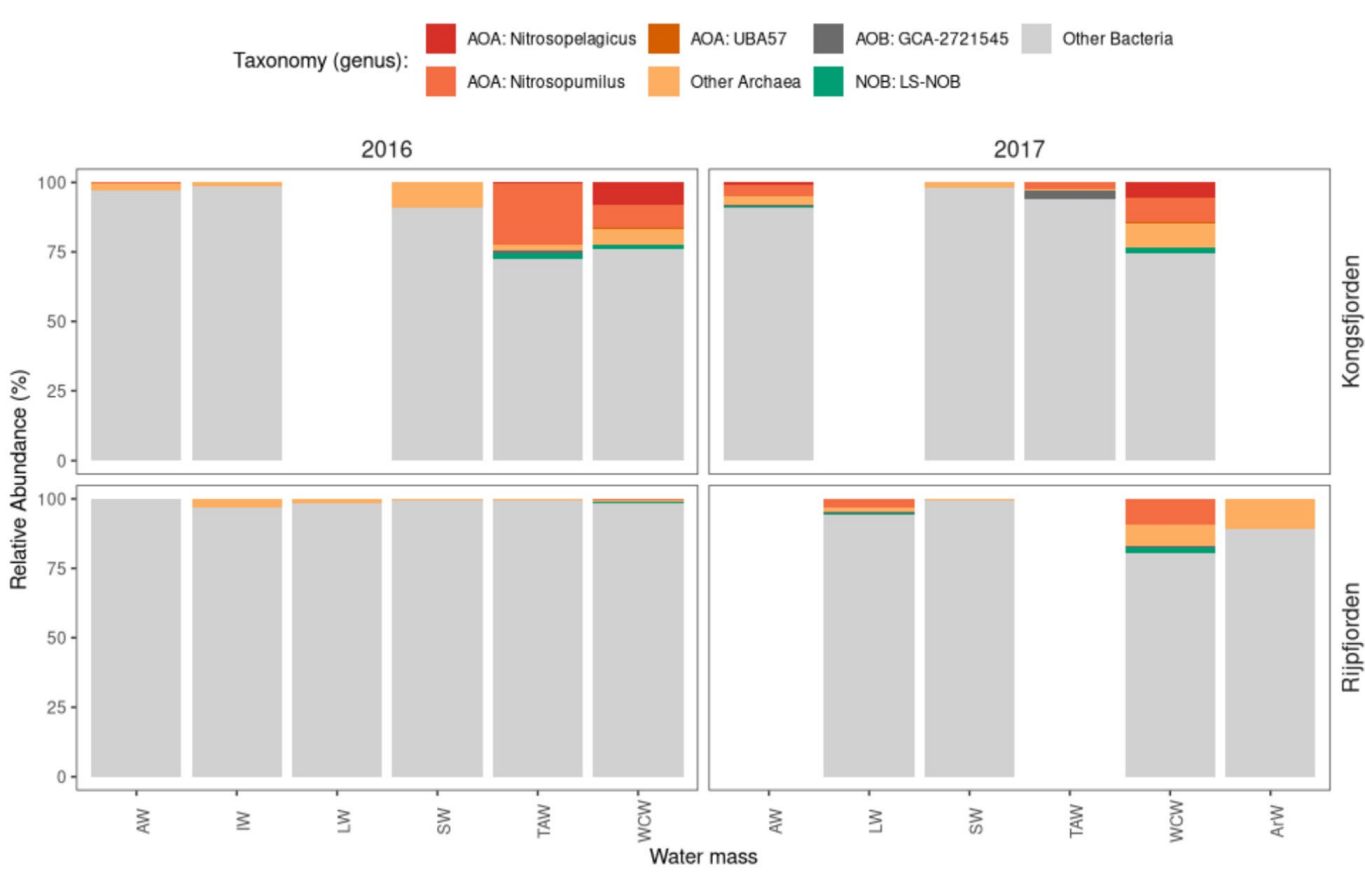
Relative abundance of genera associated with the nitrogen cycle, using V4-V5 16S rRNA gene sequencing data. Red and orange shades were used for ammonium oxidizing archaea (AOA), dark grey was used for ammonium oxidizing bacteria (AOB), green was used for nitrite oxidizing bacteria (NOB) and light grey shades were used to show the proportion of non-nitrifying archaea and bacteria. The top grids correspond to Kongsfjorden and the bottom grids correspond to Rijpfjorden; the left grids correspond to 2016, and the right grids to 2017. Blank spaces indicate the absence of a specific water mass

The taxonomic analysis from V4-V5 16S rRNA gene sequencing focusing on nitrogen (N) cycle groups showed that all analysed N cycle-related prokaryotes (ammonium and nitrite oxidizers) were more frequent at near-bottom depths (Fig. 7), reaching around 25% of the overall prokaryotic community in TAW and WCW from the Kongsfjorden transect (in both 2016 and 2017), and WCW from the Rijpfjorden transect in 2017. Differences were seen between 2016 and 2017 samples in Rijpfjorden, since these groups were more frequent in the latter year. The most abundant AOA genera were *Nitrosopumilus* and *Nitrosopelagicus*; and AOA were always more abundant than AOB and NOB (Fig. 7).

**Fig. 8:**
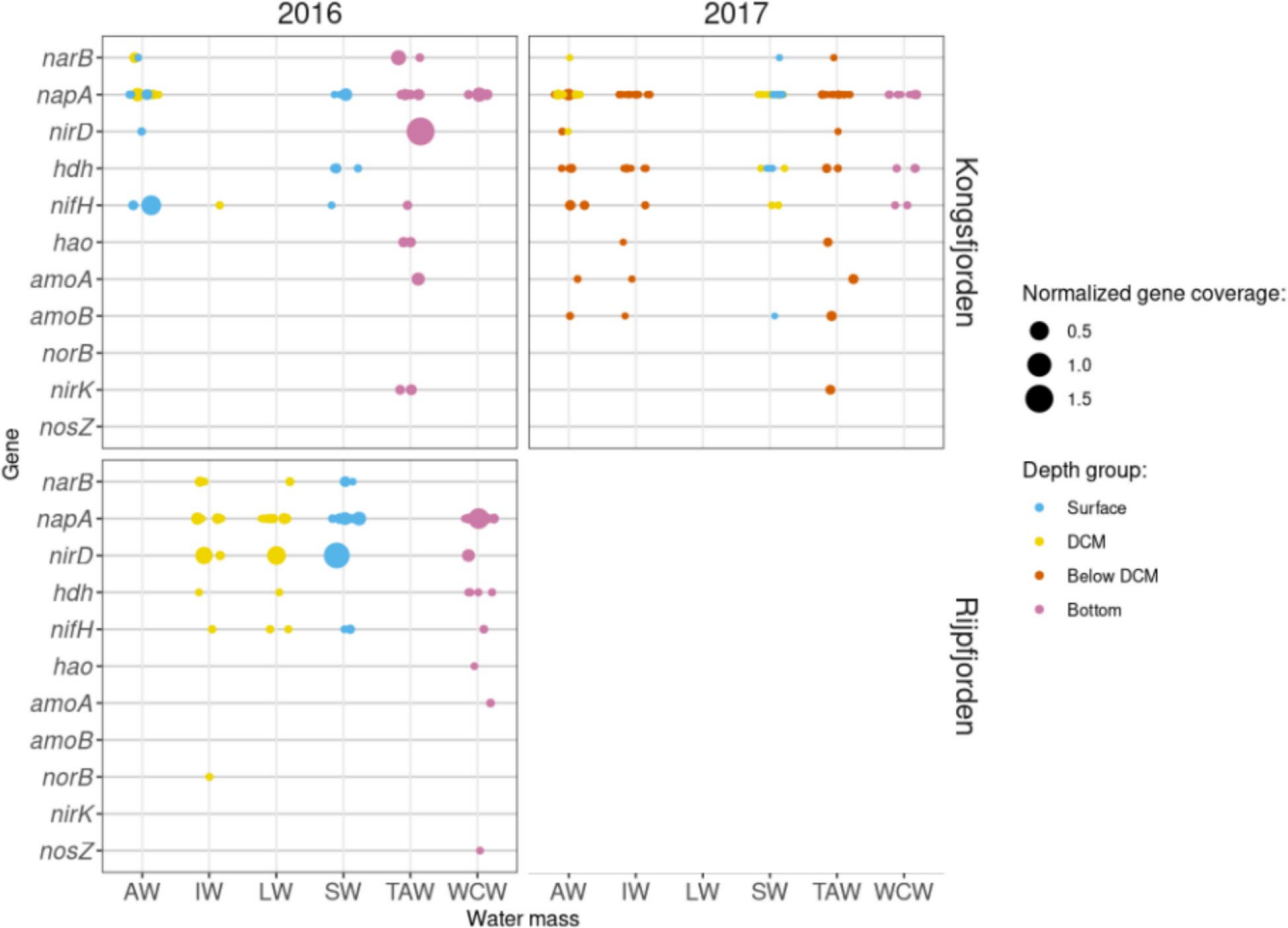
Normalized coverage of genes implicated in the nitrogen cycle, from metagenomic analysis. The top grids correspond to Kongsfjorden and the bottom grid corresponds to Rijpfjorden; the left grids correspond to 2016, and the right grids to 2017. Distinct colours denote different groups

From the functional side, the genes identified as implicated in N cycling were: *nifH* (nitrogen fixation); *amoA*, *amoB* and *hao* (nitrification); *nosZ*, *nirK* and *norB* (denitrification); *hdh* (anammox); *napA and nirD* (nitrate and nitrite reduction genes, respectively, with a role in the denitrification and dissimilatory nitrate reduction); *narB* and *nasA* (ANR—assimilatory nitrate reduction) (Fig. 8).

In 2016, the N cycle genes with higher normalized coverage were *napA* and *nirD*, with different patterns in each of the transects (Fig. 8). *napA* coverage was higher in TAW of Kongsfjorden while we observed higher coverages of *nirD* in Rijpfjorden associated mostly with IW, LW and SW (Fig. 8). Denitrification genes showed a unique distribution pattern, as different transects revealed the presence of different genes (*nirK* on Kongsfjorden; *norB* and *nosZ* on Rijpfjorden) (Fig. 8), and they were observed in low coverages and mostly associated with TAW and WCW water masses.

Results from 2017 analysis indicated a higher diversity of N-cycle genes in Kongsfjorden IW compared to 2016, when almost no genes were identified in this water mass. In 2017 an increase in the presence of nitrogen fixation (*nifH*) and anammox (*hdh*) genes was observed in most of the analysed stations of Kongsfjorden. Both 2016 and 2017, the highest gene diversity related to the nitrogen cycle was found in deep (below the deep chlorophyll maximum > 50 m) or bottom samples (Fig. 8).

## Discussion

### Hydrography and biogeochemistry

The striking difference in the fractions of AW and TAW between Kongsfjorden and Rijpfjorden reflected the increased effect of Atlantification in Kongsfjorden compared to Rijpfjorden. If we consider the hydrographic data from 2012, 2013, 2014, 2016 and 2017, we conclude that the average differences between the two fjords are expectably larger than those for 2016, which stands out as a warm year for Rijpfjorden. The cold LW and WCW masses were always found inside Rijpfjorden, dominating in all surveyed years except 2016, whereas they were not found inside Kongsfjorden. Local Water forms during autumn and winter through surface cooling and convection, whereas WCW results from convection associated with sea-ice formation [13]. Possibly, the flushing of Kongsfjorden with AW during summer dilutes the presence of these cold-water types of which WCW is where higher nutrient concentrations were measured.

Both prevalent water masses in summer 2016, AW in Kongsfjorden and TAW in Rijpfjorden, had relatively high DIN to silica (N:Si = 2.3) and low DIN to phosphorus (N:P = 13.5) atomic ratios. These values suggest that diatom growth is silicic-acid limited, given that Arctic diatoms have N:Si ratios of 0.96—1.7 (e.g., [69, 70]). Non-diatom phytoplankton are likely nitrogen-limited, considering the Redifield N:P molar ratio of 16 and nitrogen limitation in the Arctic Ocean [71]. Atlantic Water and TAW are diluted with fresh water inside the fjords leading to the formation of less dense IW and SW. These surface water masses located within the photic zone become more nitrogen depleted than AW and TAW and the excess of nitrate + nitrite relative to silicic acid may be inverted as a result of nitrogen consumption by non-diatom primary producers within the fjords and likely elevated silicic acid concentrations in glacier run-off [72, 73]. The ArW in Rijpfjorden also had a low N:P ratio reinforcing the role of nitrogen as a likely limiting factor, at least, for non-diatom phytoplankton.

The predominantly positive relationships between most nutrients and salinity shows that the sea is the main nutrient source. We found out that Kongsfjorden and Rijpfjorden (only in 2016) functioned as sinks for inorganic nitrogen, phosphate and silicic acid. This conclusion becomes only reinforced if freshwater sources in the fjords contain nutrients because the effect of dilution by freshwater is exacerbated if we assume zero nutrient concentrations, as we did, when applying Eq. 1, due to the lack of data. This conclusion aligns with the total or partial nutrient consumption in the mixed layer of Kongsfjorden and Rijpfjorden, respectively [74]. Given the greater reduction in nutrient concentrations observed in TAW from the shelf to Rijpfjorden compared to the reduction in AW entering Kongsfjorden, it is likely that nutrient sinks had a stronger impact in Rijpfjorden than in Kongsfjorden in 2016. However, it remains unclear whether this pattern reflects inherently stronger biogeochemical sinks in Rijpfjorden, or simply longer water residence time, which could enhance the cumulative effects of biogeochemical sinks [66]. While we cannot definitively identify the specific sinks involved, bacterial and phytoplankton nutrient uptake followed by cell sedimentation (the biological carbon pump) (e.g., [71]) are likely the primary sinks. The reduced nutrient sink observed in Rijpfjorden during the cold summer of 2017 co-occurs with the absence of AW and TAW and the presence of the cold and nutrient-poor ArW, pointing to a possible lower nutrient utilization.

Altogether, the results highlight both contrasts and commonalities between Kongsfjorden and Rijpfjorden, shaped by differences in water mass influence, nutrient dynamics, and freshwater input. Kongsfjorden is primarily influenced by AW and TAW, whereas Rijpfjorden is more frequently dominated by colder water masses such as Arctic ArW, LW, and WCW, with only intermittent presence of TAW. This difference likely reflects a more advanced state of Atlantification in Kongsfjorden but it also indicates that this effect spreads to Rijpfjorden during warm years. The West Spitsbergen Current (WSC), which transports AW along the continental slope west of Svalbard, exchanges water with the western fjords [13], but generally bypasses Rijpfjorden on its route in the Arctic Ocean [30] and this is the reason for the near absence of AW inside Rijpfjorden. Very likely this bypassing is a result of the shelf configuration which is much more extensive north of Rijpfjorden than west of Kongsfjorden (~ 160 versus 100 km) and density gradients resulting from the colder water in the former than in the latter fjord. However, upwelling during late winter can bring AW onto the shelf [75], where it can mix with the polar surface water and enter the fjord as TAW or Arctic Intermediate Water [30]. Such warming processes occurring early in the year can influence the summer situation in Rijpfjorden if conditions prevail through the spring. Thus, the summer conditions will then appear as a warm year, as was the case in 2016.

These patterns described above point to a complex interplay between Atlantification, water mass distribution, and biogeochemical cycling, driving distinct ecosystem dynamics in these Arctic fjords.

### Prokaryotic diversity and composition

Previous surveys in the Arctic Ocean evidenced the critical roles of water mass and geographic location in shaping prokaryote community composition [18, 20, 21, 23, 76, 77]. Our study further supports the established consensus that water masses play a pivotal role in shaping distinct prokaryotic communities, even though observed differences between water masses may be partly confounded by temporal and spatial (Kongsfjorden *versus* Rijpfjorden) variability within water masses.

Prokaryotic alpha diversity was higher in the warmer Kongsfjorden than in Rijpfjorden, partly due to the contribution of AW, and it was higher in 2017 than in 2016, in both fjords, when comparing similar water masses. This higher diversity could also be linked to differences in the timing of the phytoplankton bloom. The earlier sea ice free conditions in Kongsfjorden explains the earlier occurrence of the spring phytoplankton bloom, between late April and June [78, 79], in contrast with Rijpfjorden, where the bloom occurs in July, after the sea ice break-up [80, 81]. Earlier blooms create a longer and more dynamic period of phytoplankton-driven resource availability, generating multiple ecological niches that support greater microbial diversity [18]. In contrast, Rijpfjorden’s later bloom results in a shorter and less variable production season, limiting resource-availability pulses and niche diversification [18].

Alpha diversity increased in deeper water masses, reinforcing the idea that depth can covary positively with microbial diversity [82]. This is in accordance with studies from the Pacific [39], the North Atlantic [83, 84] and the Mediterranean Sea [85]. Very likely, this covariation results from other depth-dependent factors. The higher nutrient concentrations at depth, resulting from the remineralization of the sinking organic matter, such as we observed in TAW and WCW, and the proximity to the marine sediments, may be related to the observed increased diversity (e.g., [86, 87]).

The deepest samples, taken from WCW, had similar prokaryotic communities between both study areas (in Kongsfjorden we found this water mass only in the deep Fram Strait, Fig. S4). This water mass had a very specific prokaryotic signature which was preserved at ~ 1000 m in the open ocean areas of both Rijpfjorden and Kongsfjorden transects (R8 and V6 stations, respectively). Evidence from the Arctic Ocean indicates that deep water masses, such as WCW, can retain highly characteristic prokaryotic communities during transport across basins. Studies of meso- and bathypelagic waters in the Fram Strait demonstrate that deep Arctic water masses harbour stable and characteristic prokaryotic assemblages whose composition remains consistent across distant locations, reflecting their physical isolation and long residence times [76]. Similar patterns have been reported for chemolithoautotrophic groups such as Thaumarchaeota (Nitrososphaeria), whose deep-water populations track the distribution of specific water masses rather than geographic proximity [20]. Recent work further shows that the vertical stratification of bacterial and archaeal communities in the Arctic is strongly governed by deep water mass identity, with comparable assemblages occurring wherever these layers are present [77]. Collectively, these findings support the interpretation that WCW can preserve a distinct prokaryotic signature that is transported and preserved over large spatial scales.

Notable differences were observed in the relative abundances of the main prokaryotic phyla identified in both fjords. Proteobacteria (with *Pelagibacter* being the dominant genus) was the most abundant phylum in Kongsfjorden, in accordance with what has been described in previous surveys in this fjord and other Arctic Ocean regions [77, 88, 89].The *Pelagibacter* genus belongs to the former SAR11 clade, which is one of the most dominant groups found in the oceans [90]. The SAR11 clade was found in AW in Isfjorden (fjord further south on the west coast of Spitsbergen), during a summer survey [91]. Genomic analysis of *Pelagibacter* has revealed genes adapted to grow under oligotrophic conditions [92], such as post-phytoplankton bloom stages, when a shift from copiotrophic to oligotrophic communities is usually observed [77].

A study in different regions of Svalbard has shown that western and northern fjords show significant differences among prokaryotic communities [89]. High relative abundances of Bacteroidota and Verrucomicrobiota were recorded in Raudfjorden, a fjord located in the northern region of Svalbard [89], similarly to what we found in the Rijpfjorden transect. This unique bacterial signature among different Svalbard fjords is thought to be related to the influence of different water masses and hydrographic conditions [93–97]. A study from Dease Strait (NW Canada) during the spring phytoplankton bloom, indicated that bacterial clades specialized in polysaccharide metabolism, such as Bacteroidota, became dominant as the phytoplankton bloom progressed displacing *Archaea*, Alphaproteobacteria, and Gammaproteobacteria [98]. ​Similarly to Bacteroidota*,* the phylum Verrucomicrobiota is also known for the degradation of diverse polysaccharides and the prevalence of these phyla suggests their role in the breakdown of organic compounds from the spring blooms in Arctic regions [1]. The time difference of these blooms may explain part of the prokaryotic community differences between both fjords.

Many Thermoproteota species play a crucial role in the nitrogen cycle, particularly in ammonium oxidation (*e.g. Nitrosopumilus* and other ammonium-oxidizing Archaea) [99]. The higher relative abundance of nitrifiers and amoA genes at deeper stations indicate an increased genetic potential for nitrification but does not provide evidence of active nitrification in the absence of gene expression or rate measurements. This pattern is, however, consistent with the elevated NO₃⁻ concentrations measured at deeper stations. Previous studies pointed towards protective behaviour by nitrifiers against the effect of photochemically-produced reactive oxygen species in surface waters [100]. Other studies in the Arctic have reported the presence of nitrifiers in surface waters during the polar night, with a marked decline in early summer [21] and observed that deeper water layers were dominated by *Candidatus nitrosopumilus* [101]. These findings align with our results, which show that Thaumarchaeota (Nitrososphaeria) accounts for approximately 25% of the prokaryotic community in deeper water stations. Among the nitrifiers, AOA were more abundant than AOB and NOB, which is consistent with studies from the North Atlantic [83, 84]. Previous studies in the Arctic Ocean have also associated NOB and AOA distributions with specific water masses, besides depth [20]. In our data, AOA were more abundant in some TAW stations and WCW waters of Kongsfjorden and Rijpfjorden, suggesting that these prokaryotes are mostly dependent on depth-related factors rather than on specific water masses, although water mass characteristics might influence the ecotypes of nitrifiers [20, 102]. This distribution pattern of the nitrifiers among the different water masses also aligns with the previously discussed data on nutrient concentrations in the different transects and water masses, namely with low ammonium and high nitrate + nitrite values in samples with higher relative abundances of AOB/AOA and NOB (Table 1, S6 and Fig. 7). The enrichment of nitrification (*amoA*, *amoB* and *hao*) and denitrification genes (*nirK*, *norB* and *nosZ*) in stations dominated by IW, TAW, and WCW, was corroborated by V4-V5 16S rRNA gene sequencing, and highlights the specialized microbial communities adapted to the unique conditions of these deep environments in Arctic waters [20].

Denitrification genes (*nirK* and *nosZ*) were identified in both transects, mostly with low coverage levels, except for a *nirK* hotspot (observed in the bottom of some Kongsfjorden shelf and coastal stations), and a *nosZ* hotspot (identified in Rijpfjorden in 2016). Low denitrification gene coverage was expected, since the process occurs in sub-oxic or anoxic environments and it is known to be more efficient in sediments [71, 103]. However, our results indicate a potential for sedimentary denitrification in the bottom waters of Kongsfjorden stations, particularly in the deeper water masses enriched with nitrates, such as TAW and WCW. At the same time, we observed in these water masses a high relative abundance of nitrifiers, from the genera *Nitrosopelagicus* and *Nitrosopumilus*. In contrast, nitrification or denitrification potential were not observed in shallower stations. Our results provide supporting evidence for the occurrence of nitrogen fixation in the surface waters. The nitrogen fixation gene *nifH* was identified in both transects, but with higher coverage in AW of Kongsfjorden. A recent work conducted in Kongsfjorden has shown that diazotrophs carrying *nifH* gene are present in this fjord system, with communities being structured by water masses and dominated by non-cyanobacterial Clusters I and III phylotypes [104]. This study further reports several *nifH* OTUs enriched in Atlantic-influenced waters, supporting our observation of higher *nifH* signal in AW and indicating that nitrogen-fixing potential in Svalbard fjords is tightly coupled to hydrographic regimes. Additionally, *nifH* genes have also been detected in Cyanobacteria and/or heterotrophs in the Fram Strait and Greenland Sea collected from superficial matrices, including waters, sea ice and melt ponds [105–107]. Assimilatory nitrate reduction (ANR, *narB* and *nasA*) displayed low coverages, but *napA* and *nirD* presented high coverages in specific stations of both transects in 2016, but with different patterns. Given that these genes are involved in both dissimilatory nitrate reduction to ammonia (DNRA) and denitrification pathways [e.g., *napA* in the conversion of NO₂ to NO₃ in both processes; *nirD* in the reduction of nitrite to nitric oxide (NO) during denitrification and to ammonia in DNRA], their higher prevalence in TAW of Kongsfjorden in 2016 may be associated with the previously discussed denitrification potential of bottom water masses, rather than with DNRA. Studies focused on *nirD* abundance in the Arctic are very scarce, with a few reporting the presence of this gene, at low rates, in the sediments of the Chukchi Sea [108] and in Kongsfjorden and Smeerenburgfjorden in Svalbard [109]. Regarding Arctic waters, recent findings have indicated that *nirD* abundance was higher in Kongsfjorden samples, than shelf stations [110]. The role of DNRA in marine microorganisms remains poorly understood; however, it is anticipated to outcompete denitrification when electron donors are more abundant than nitrate [111]. In Rijpfjorden, the higher coverage of *nirD* suggests that nitrogen retention may be occurring via the DNRA pathway, further supported by the lower abundance of nitrogen-fixing gene (*nifH*), which can be an indicator of a more limited nitrogen input in this environment. Nutrient concentrations also support this trend since ammonium levels were consistently lower in Rijpfjorden than Kongsfjorden (Tables 1 and S6). DNRA is an anaerobic respiration process that is known mainly from prokaryotic organisms, although previous stable isotope experiments demonstrated the occurrence of dissimilatory reduction of NO_3_^−^ by benthic and pelagic diatoms [112]. These facts can also explain the occurrence of *nirD* genes in our metagenomic data where diatoms have been shown to be a prevalent group of Kongsfjorden and Rijpfjorden phytoplankton [30].

Since our functional assessment was based on genomic-level methods, we can only indicate the existence of genes implicated in nitrogen cycling, but we cannot assume up-/down-regulation or activity. Future studies with deeper sequencing could provide a more comprehensive analysis of the genetic potential for nitrogen cycling. Additionally, incorporating alternative omics approaches (such as metatranscriptomics or metaproteomics) and measuring rates of nitrogen-related pathways could offer greater insight into the actual activity of these microorganisms.

## Conclusions

We have shown that Kongsfjorden and Rijpfjorden have distinct hydrographic regimes, resulting largely from the larger impact of Atlantification in Kongsfjorden. Our observations indicate that warm Atlantic Water (AW) consistently dominated Kongsfjorden in both study years. In contrast, Rijpfjorden was primarily influenced by cooler Transformed Atlantic Water (TAW) in the relatively warm year 2016, and by colder Arctic Water (ArW) and Winter-Cooled Water (WCW) in the other years. Konsgfjorden acted as a summer nutrient sink, whereas Rijpfjorden showed similar behaviour only in 2016. This suggests that warmer water masses are linked to higher nutrient uptakes. These hydrographic and biogeochemical differences were further supported by the distinct prokaryotic communities observed in each fjord, with major changes in the diversity and community structure, shaped by water mass types and their depth ranges. The presence of Atlantic Water (AW) appears to have a strong influence on microbial community structure, providing valuable insights into how Atlantification may be driving shifts in microbial assemblages and functions. We found higher prokaryotic diversity in Kongsfjorden, and higher abundance of Proteobacteria and Thermoproteota in Kongsfjorden and Bacteroidota in Rijpfjorden. Our metagenomic analysis showed distinct nitrogen functional profiles between Rijpfjorden and Kongsfjorden, with Kongsfjorden being characterized by higher coverage of diazotrophs associated genes within the AW. In both transects, intermediate and deeper water masses (IW, TAW and WCW) seem to be important pools for genetic diversity associated with the nitrogen cycle. This includes ammonium- and nitrite-oxidizing prokaryotes (NOB), which were most abundant in bottom-associated waters—particularly TAW and WCW in the Kongsfjorden transect during both 2016 and 2017, and WCW in Rijpfjorden in 2017. In these layers, their relative abundance reached up to 25% of the microbial community, predominantly composed of archaeal taxa (AOA), mainly from the genera *Nitrosopumilus* and *Nitrosopelagicus*.

## Supplementary Information

Supplementary Material 1.

Supplementary Material 2: Table S1.

Supplementary Material 3: Table S2. 

Supplementary Material 4: Table S3.

## Data Availability

The datasets supporting the conclusions of this article are available in the following repositories: All FASTQ files relative to the V4-V5 16S rRNA gene amplicon and shotgun metagenomics sequencing are publicly available at the European Nucleotide Archive, under accession number: PRJEB24517 (MOSJ2016) and PRJEB72025 (MOSJ2017) ([https://www.ebi.ac.uk/ena/browser/view/PRJEB24517] (https://www.ebi.ac.uk/ena/browser/view/PRJEB24517)). Species abundance tables after processing the raw reads from the above dataset: [https://doi.org/10.21334/NPOLAR.2024.E9FD36A1] (https://doi.org/10.21334/NPOLAR.2024.E9FD36A1) Ocean physical data (CTD profiles): [https://doi.org//10.21334/NPOLAR.2024.7414766F] (https://doi.org/10.21334/NPOLAR.2024.7414766F). Ocean biogeochemical data: https://doi.org/10.21334/NPOLAR.2024.4D4DE169. No special codes were developed for this study.
