# Supplementary material for: Comparative analysis of prokaryotic communities, hydrography, and biogeochemistry in Atlantic vs non-Atlantic influenced Svalbard fjords
Source: BMC Microbiol. 2026 Feb 27;26:301. doi: 10.1186/s12866-026-04821-2 (PMC13041043; doi:10.1186/s12866-026-04821-2)
Supplement: Supplementary file 1 — Supplementary Material 1. [file 12866_2026_4821_MOESM1_ESM.docx]

**Journal:** [BMC](https://environmentalmicrobiome.biomedcentral.com/) Microbiology

Supporting Information for

**Comparative Analysis of Procaryotic Communities, Hydrography, and Biogeochemistry in Atlantic vs non-Atlantic Influenced Svalbard Fjords**

Joana Costa^1^, Francisco Pascoal^1,2^, Mafalda S. Baptista^1^, Haakon Hop^3^, Philipp Assmy^3^, Anette Wold^3^, Catarina Magalhães^1,2^* and Pedro Duarte^3^*

^1^ Interdisciplinary Centre of Marine and Environmental Research, University of Porto, Terminal de Cruzeiros do Porto de Leixões, Av. General Norton de Matos s/n, 4450-208 Porto, Portugal;

^2^ Departamento de Biologia, Faculdade de Ciências, Universidade do Porto, rua do Campo Alegre s/n, 4169– 007 Porto, Portugal;

^3^ Norwegian Polar Institute, Fram Centre, N-9296 Tromsø, Norway;

*Corresponding authors:

Catarina Magalhães (catarina.magalhaes@fc.up.pt)

Pedro Duarte (Pedro.Duarte@npolar.no)

**Contents of this file**

- Text S1
- Supplementary Figures S1-S26
- Supplementary Tables S4-S13. Tables S1, S2 and S3 are provided in separate files given their large size.

**Introduction**

This file includes results from genomic and metagenomic reads (Text S1) and supplementary figures and tables. Some tables do not fit inside a word document (S1-S3), but the original files were provided for peer review in the submission platform. All figure and table captions are available in the original manuscript file.

**Text S1**

Sequencing generated between 83 431 and 136 227 raw reads from V4-V5 16S rRNA gene amplicons (mean = 46 093, sd = 25 797, n = 56), and the number of high-quality reads assigned to prokaryotic taxa varied from 6 301 to 86 845 per sample (mean = 31 448 reads, sd = 17 602, n = 56). Thus, among the samples analyzed, from 53.9 to 75.8% of raw reads were retained during the quality filtering procedures (Table S2). From the shotgun metagenomic sequencing, we initially obtained between 3 184 673 and 1 735-2 259 raw reads (mean = 9 895 368, sd = 3 336 266, n = 40), and the percentage of Q30 bases remaining after the trimming was between 81 and 94% (Q30 base number mean =1 038 156 764, sd = 389 670 228, n = 44) (Table S3).

**Supplementary Figures**


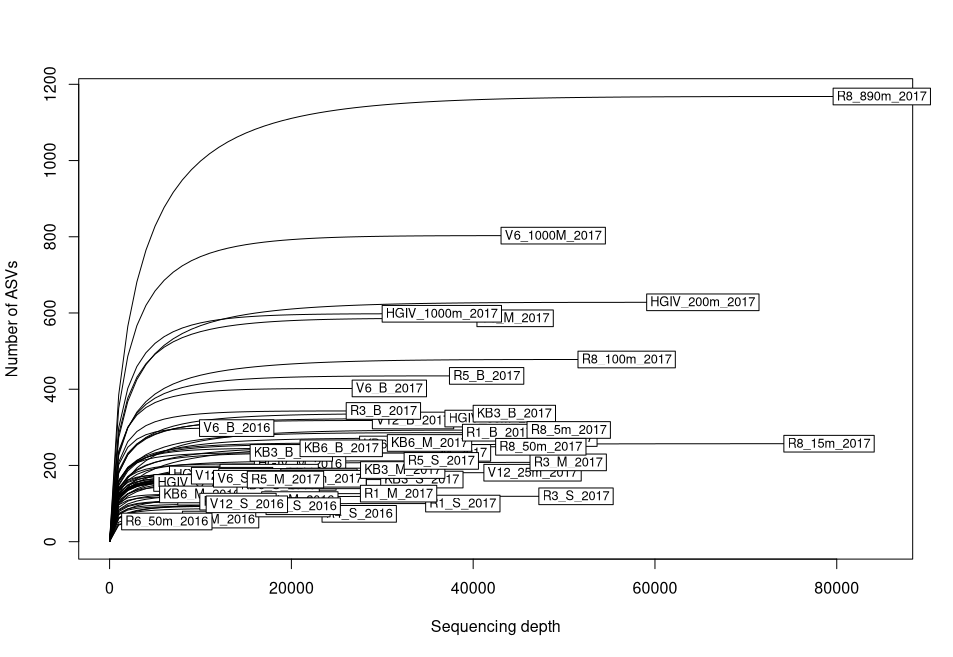


**Fig. S1** Rarefaction curves of sequencing results from V4-V5 16S rRNA gene amplicons and shotgun metagenomics.


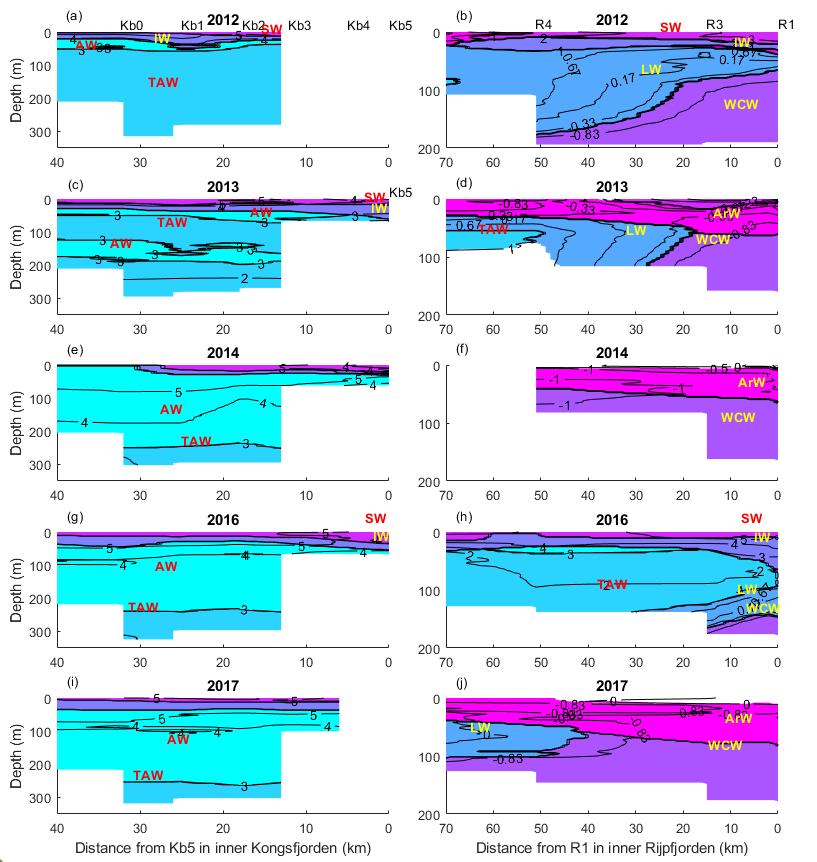


**Fig. S2** Isotherms (conservative temperature) and water masses [labels in the figure, Arctic Water (ArW), Atlantic Water (AW), Intermediate Water (IW), Local Water (LW), Surface Water (SW), Transformed Atlantic Water (TAW) and Winter-cooled Water (WCW)] in Kongsfjorden (left panels) and in Rijpfjorden (right panels) in July-August 2012, 2013, 2014, 2016 and 2017. Refer Fig. 1, Table S5 and Methods, Sampling collection.


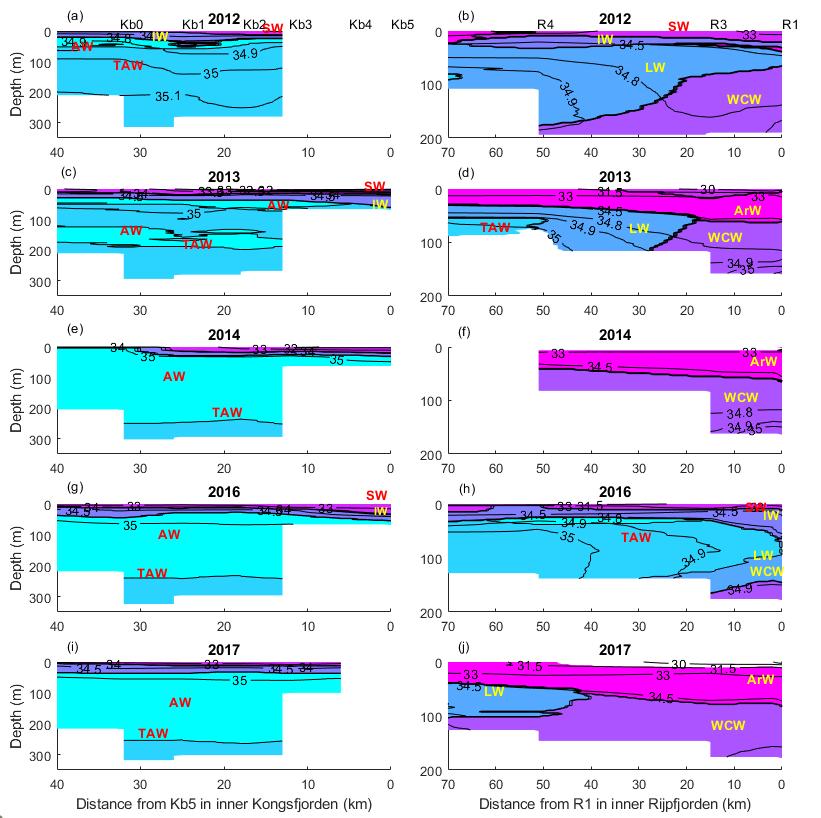


**Fig. S3** Isohalines (absolute salinity) and water masses [labels in the figure, Arctic Water (ArW), Atlantic Water (AW), Intermediate Water (IW), Local Water (LW), Surface Water (SW), Transformed Atlantic Water (TAW) and Winter-cooled Water (WCW)] in Kongsfjorden (left panels) and in Rijpfjorden (right panels) in July-August 2012, 2013, 2014, 2016 and 2017. Refer Fig. 1, Table S5 and Methods, Sampling collection.


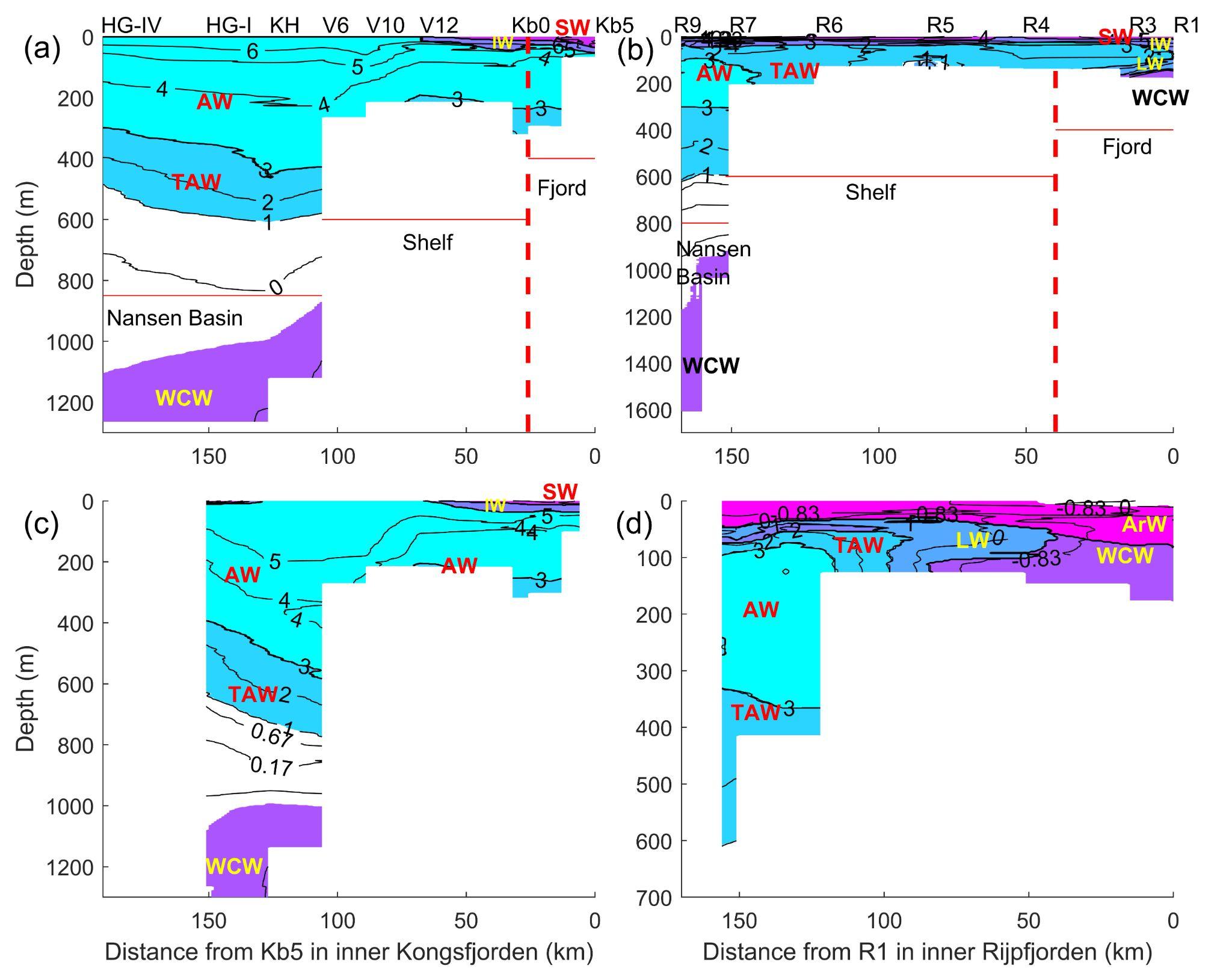


**Fig. S4** (a, b) Isotherms (conservative temperature) and water masses [labels in the figure, Arctic Water (ArW), Atlantic Water (AW), Intermediate Water (IW), Local Water (LW), Surface Water (SW), Transformed Atlantic Water (TAW) and Winter-cooled Water (WCW)] along the Kongsfjorden and the Rijpfjorden transects in the summer of 2016, respectively. (c, d) like the upper panels but for the summer of 2017. The vertical red dashed lines mark the mouth of the fjords. The extension of the fjord, shelf and deep Nansen Basin are shown by the red horizontal lines. The white areas in the deep basins correspond to depth ranges with few data points to classify the water masses. Refer Fig. 1, Table S5 and Methods, Sampling collection.


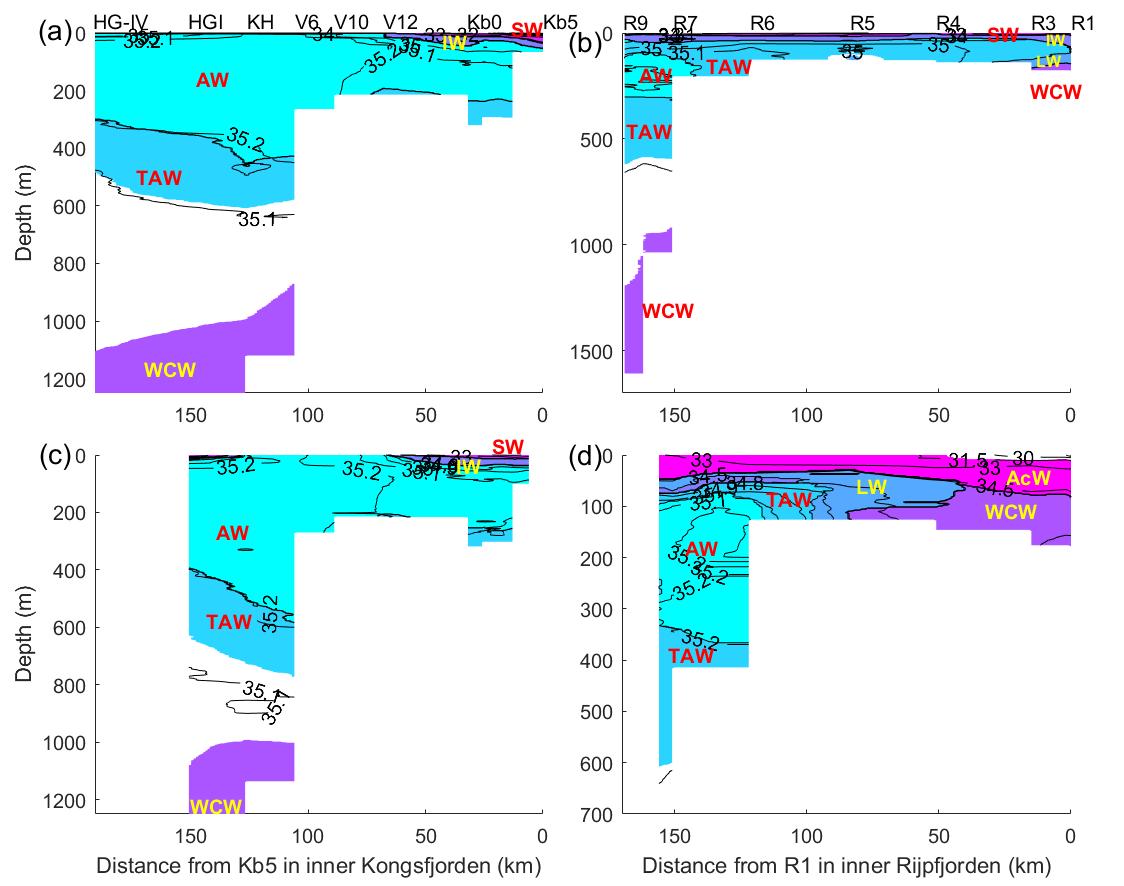


**Fig. S5** (a, b) Isohalines (absolute salinity) and water masses [labels in the figure, Arctic Water (ArW), Atlantic Water (AW), Intermediate Water (IW), Local Water (LW), Surface Water (SW), Transformed Atlantic Water (TAW) and Winter-cooled Water (WCW)] along the Kongsfjorden and the Rijpfjorden transects in the summer of 2016, respectively. (c, d) like the upper panel but for the summer of 2017. The white areas in the deep basins correspond to depth ranges with few data points to classify the water masses. Refer Fig. 1, Table S5 and Methods, Sampling collection.


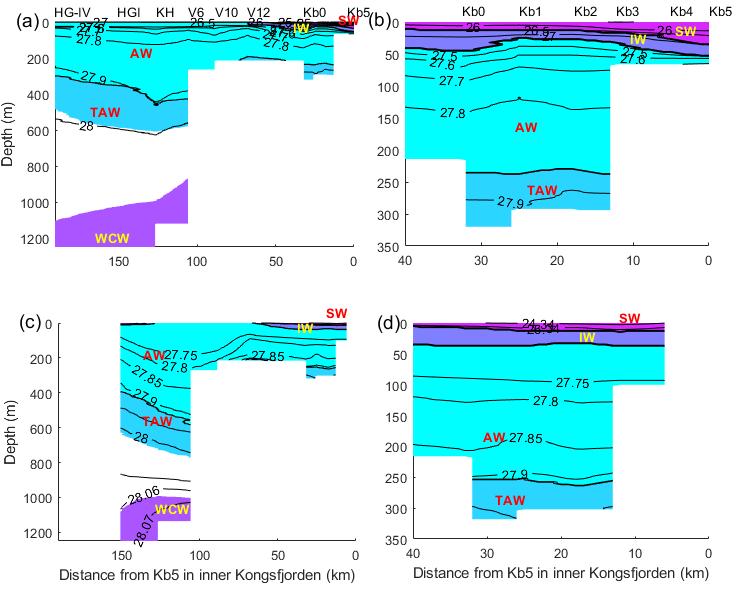


**Fig. S6** Isopycnic (sigma) lines and water masses [labels in the figure, Atlantic Water (AW), Intermediate Water (IW), Surface Water (SW), Transformed Atlantic Water (TAW) and Winter-cooled Water (WCW)] along the Kongsfjorden transect. (a, c) the full transect for 2016 and 2017, respectively; (b, d) detail of the transect between the inner fjord and beginning of the shelf, for 2016 and 2017, respectively. Refer Fig. 1, Table S5 and Methods, Sampling collection.

**
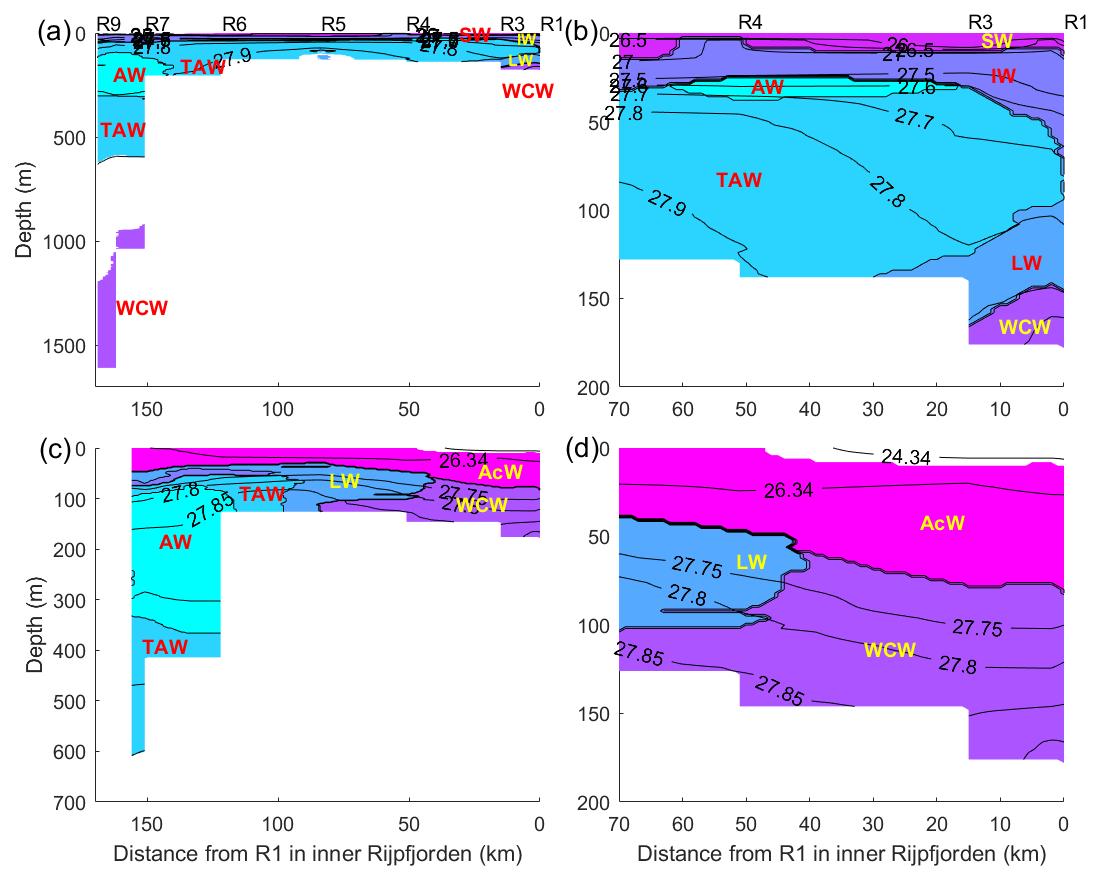
**

**Fig. S7** Isopycnic (sigma) lines and water masses [labels in the figure, Arctic Water (ArW), Atlantic Water (AW), Intermediate Water (IW), Local Water (LW), Surface Water (SW), Transformed Atlantic Water (TAW) and Winter-cooled Water (WCW)] along the Rijpfjorden transect. (a, c) the full transect for 2016 and 2017, respectively; (b, d) detail of the transect between the inner fjord and beginning of the shelf, for 2016 and 2017, respectively. Refer Fig. 1, Table S5 and Methods, Sampling collection.


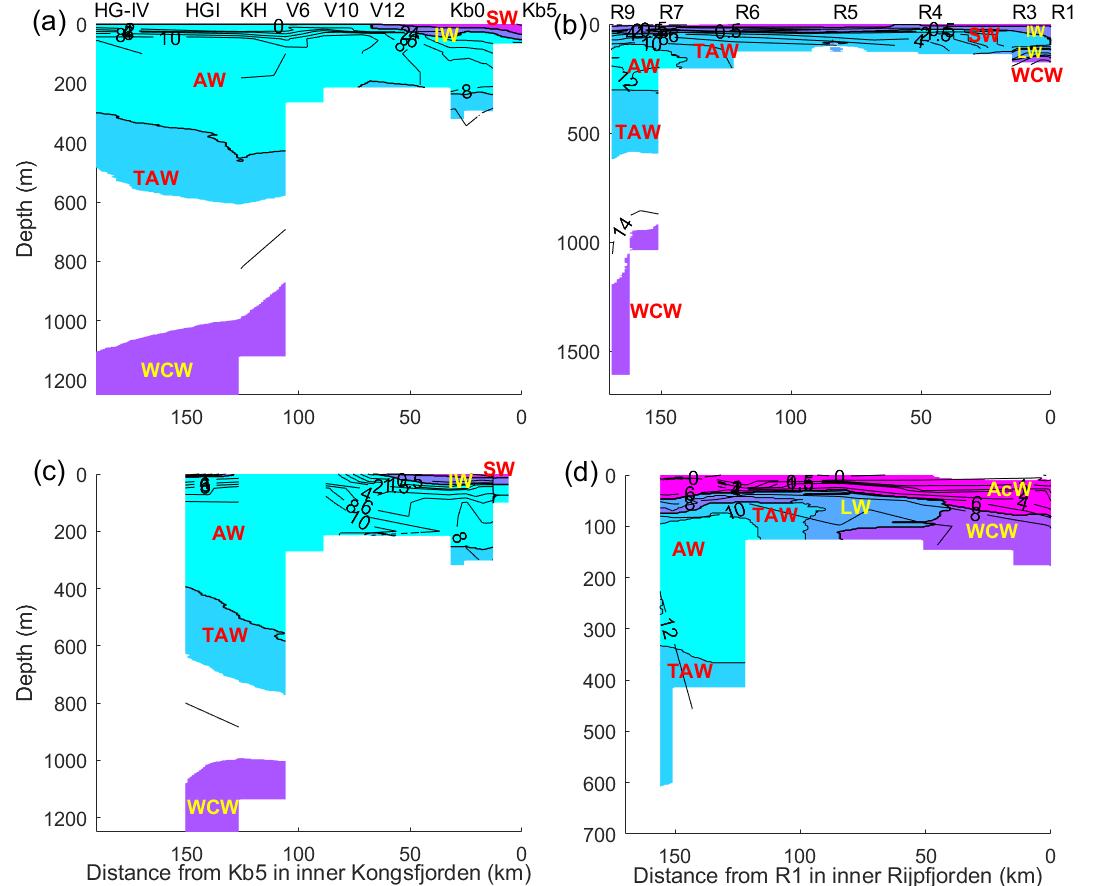


**Fig. S8** (a, b) Nitrate + nitrite concentration isolines (mmol m^-3^) and water masses [labels in the figure, Arctic Water (ArW), Atlantic Water (AW), Intermediate Water (IW), Local Water (LW), Surface Water (SW), Transformed Atlantic Water (TAW) and Winter-cooled Water (WCW)] along (a) the Kongsfjorden transect and (b) the Rijpfjorden transect in July 2016, respectively. (c, d) the same as the upper panels but for 2017. The white areas in the deep basins correspond to depth ranges with few data points to classify the water masses. Refer Fig. 1, Table S5 and Methods, Sampling collection.

**
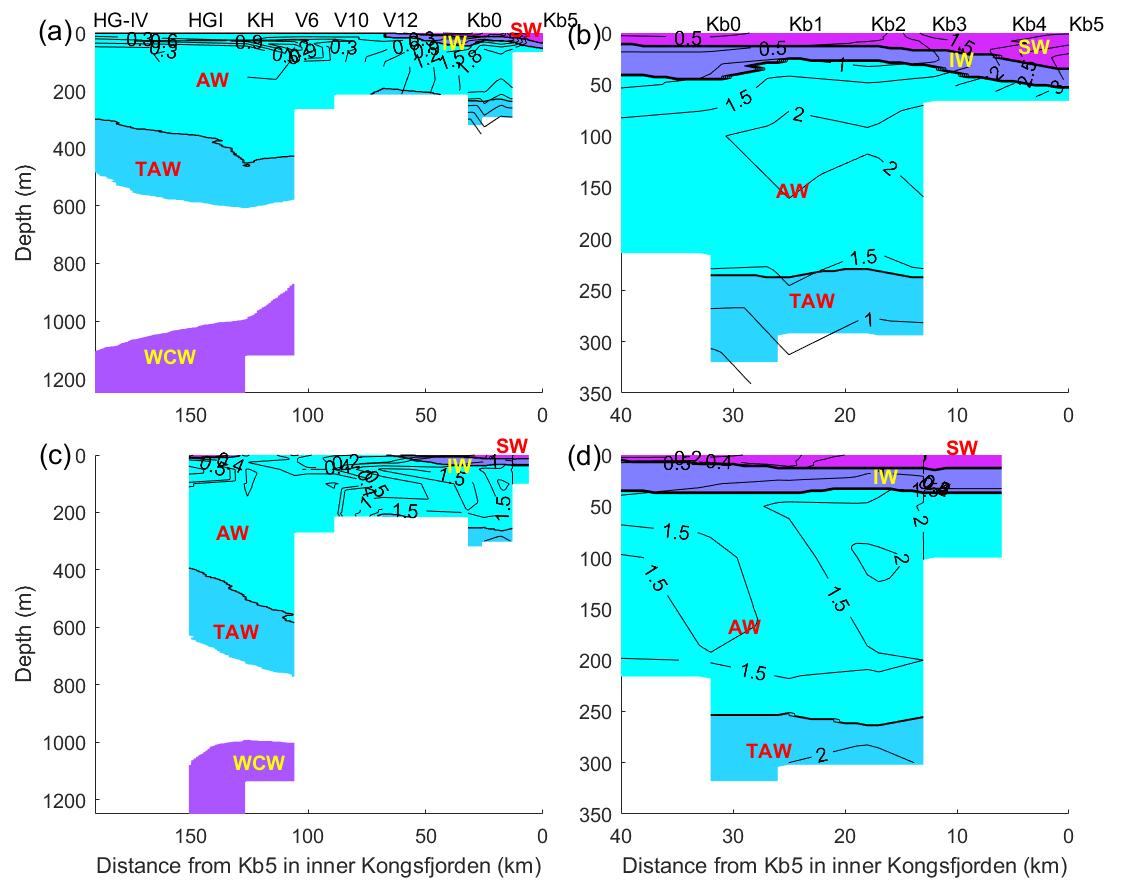
**

**Fig. S9** Ammonium concentration isolines (mmol m^-3^) lines and water masses [labels in the figure, Atlantic Water (AW), Intermediate Water (IW), Surface Water (SW), Transformed Atlantic Water (TAW) and Winter-cooled Water (WCW)] along the Kongsfjorden transect. (a, c) the full transect for 2016 and 2017, respectively; (b, d) detail of the transect between the inner fjord and beginning of the shelf, for 2016 and 2017, respectively. Refer Figure 1, Table S5 and Methods, Sampling collection.

**
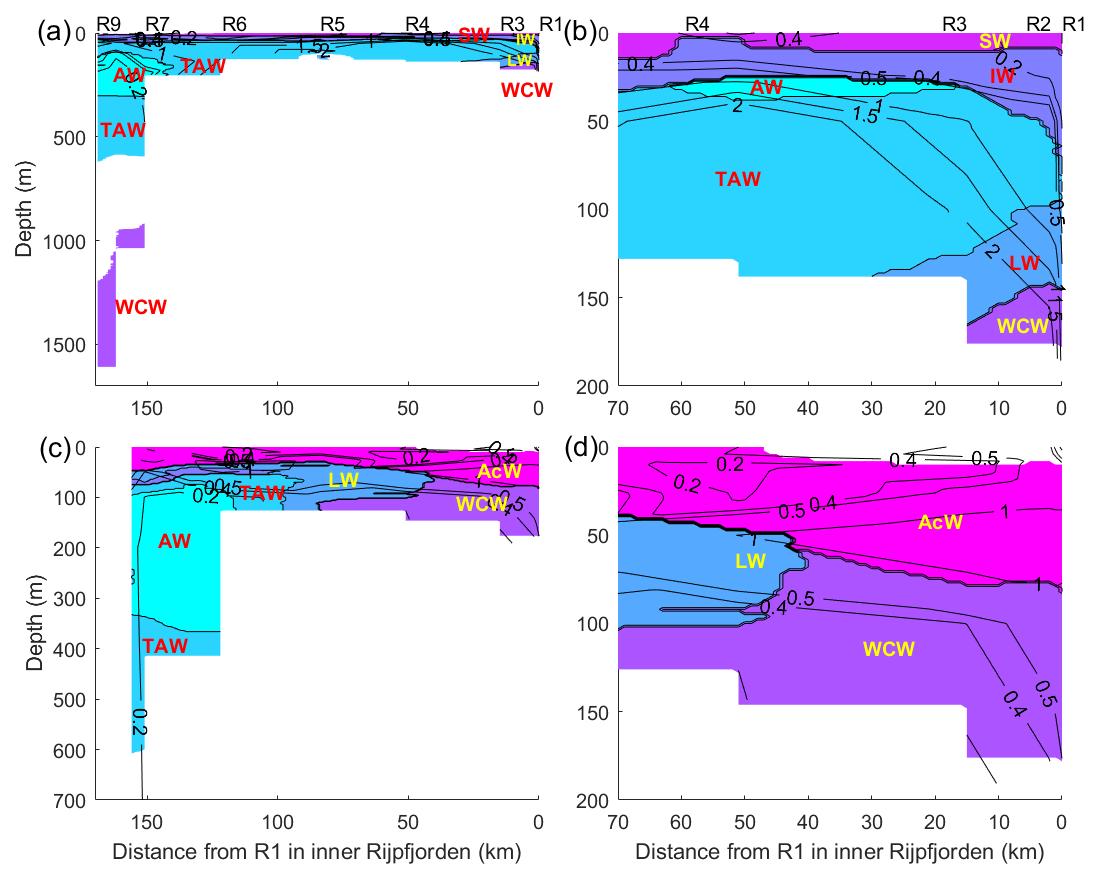
**

**Fig. S10** Ammonium concentration isolines (mmol m^-3^) and water masses [labels in the figure, Arctic Water (ArW), Atlantic Water (AW), Intermediate Water (IW), Local Water (LW), Surface Water (SW), Transformed Atlantic Water (TAW) and Winter-cooled Water (WCW)] along the Rijpfjorden transect. (a, c) the full transect for 2016 and 2017, respectively; (b, d) detail of the transect between the inner fjord and beginning of the shelf, for 2016 and 2017, respectively. Refer Fig. 1, Table S5 and Methods, Sampling collection.

**
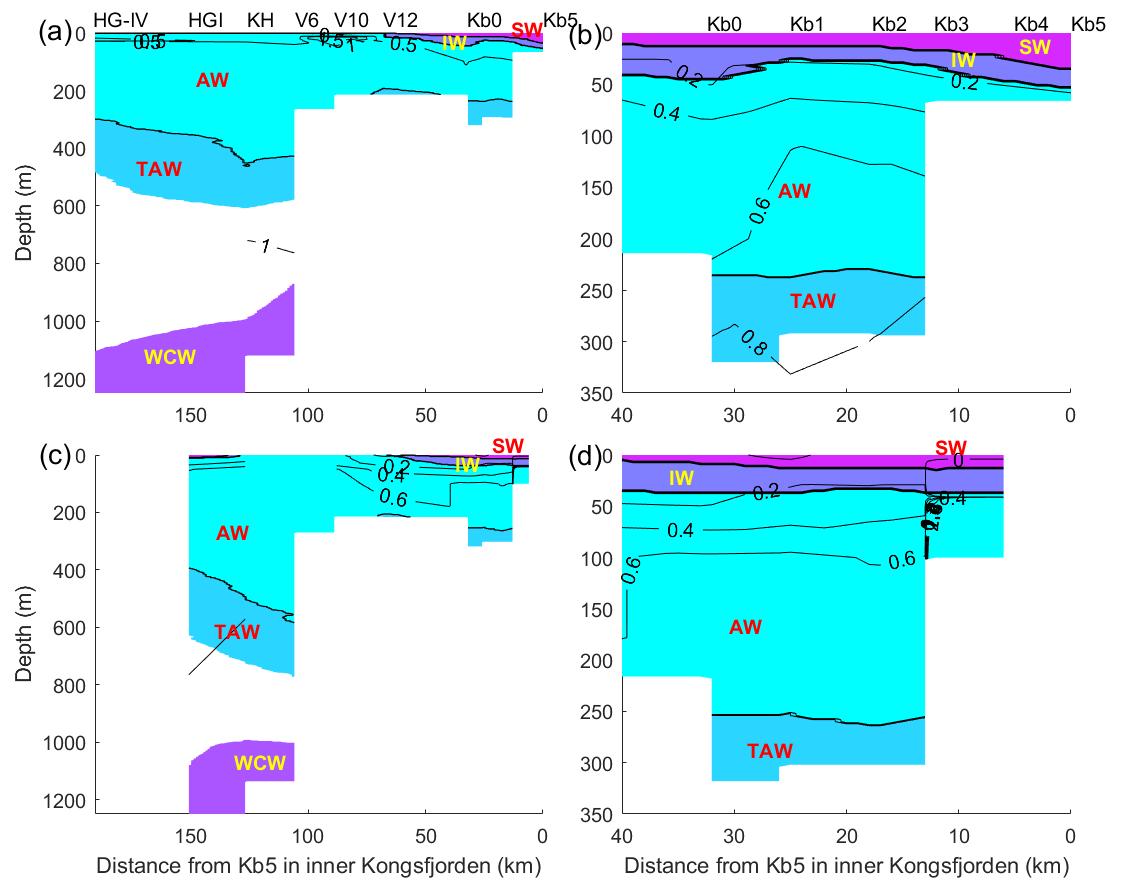
**

**Fig. S11** Phosphate concentration isolines (mmol m^-3^) and water masses [labels in the figure, Atlantic Water (AW), Intermediate Water (IW), Surface Water (SW), Transformed Atlantic Water (TAW) and Winter-cooled Water (WCW)] along the Kongsfjorden transect. (a, c) the full transect for 2016 and 2017, respectively; (b, d) detail of the transect between the inner fjord and beginning of the shelf, for 2016 and 2017, respectively. Refer Fig. 1, Table S5 and Methods, Sampling collection.

**
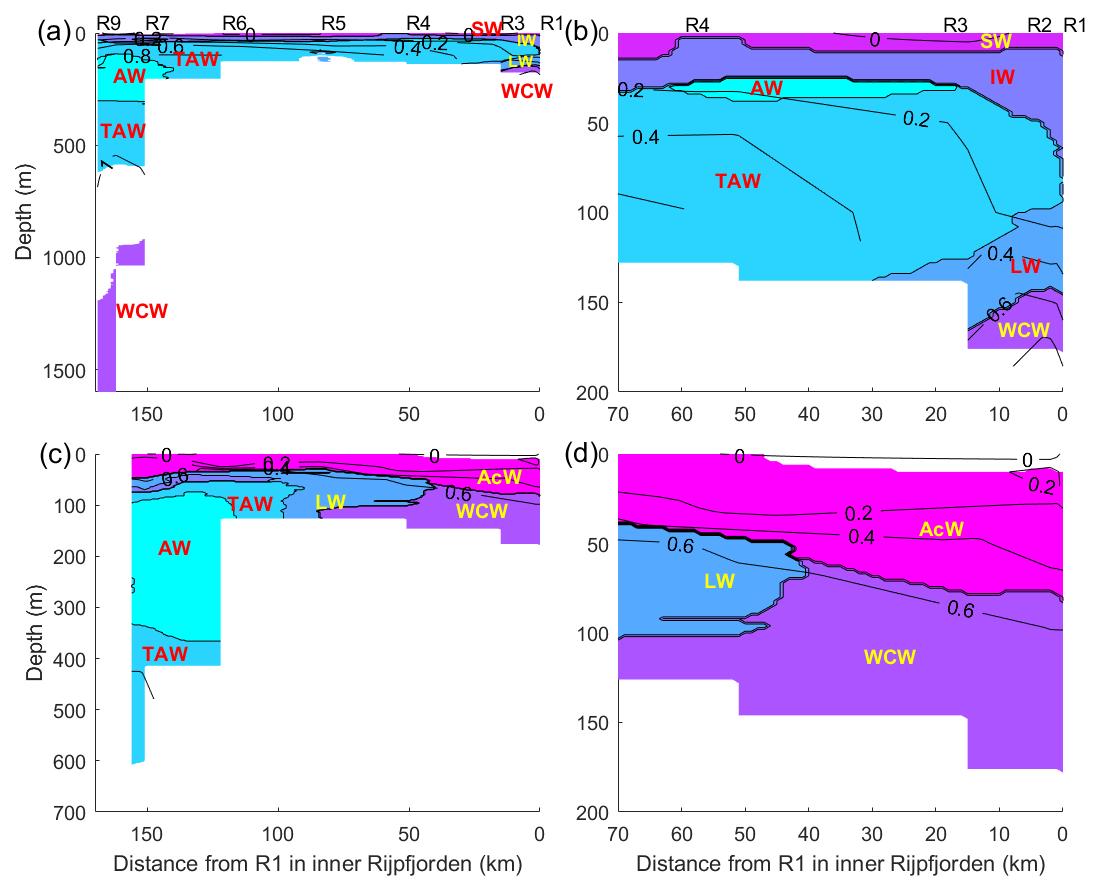
**

**Fig. S12** Phosphate concentration isolines (mmol m^-3^) and water masses [labels in the figure, Arctic Water (ArW), Atlantic Water (AW), Intermediate Water (IW), Local Water (LW), Surface Water (SW), Transformed Atlantic Water (TAW) and Winter-cooled Water (WCW)] along the Rijpfjorden. (a, c) the full transect for 2016 and 2017, respectively; (b, d) detail of the transect between the inner fjord and beginning of the shelf, for 2016 and 2017, respectively. Refer Fig. 1, Table S5 and Methods, Sampling collection.

**
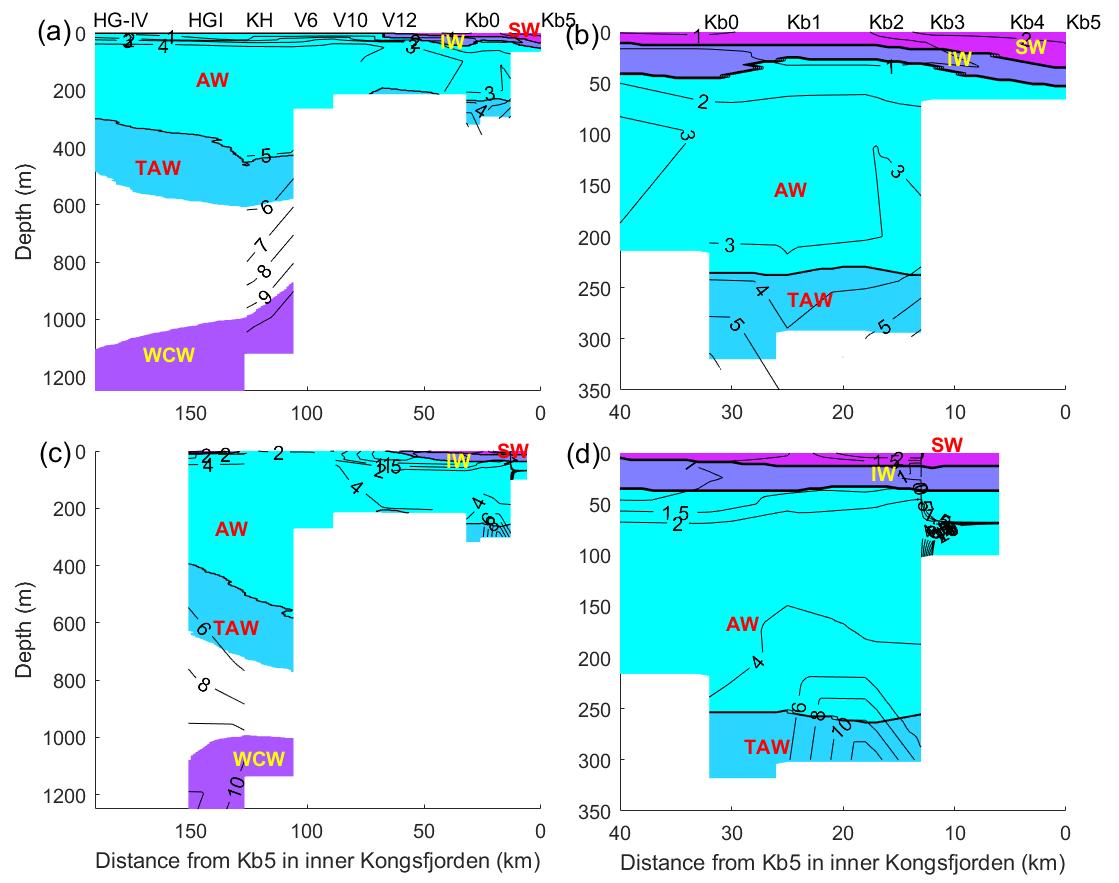
**

**Fig. S13** Silicic acid concentration isolines (mmol m^-3^) and water masses [labels in the figure, Atlantic Water (AW), Intermediate Water (IW), Surface Water (SW), Transformed Atlantic Water (TAW) and Winter-cooled Water (WCW)] along the Kongsfjorden transect. (a, c) the full transect for 2016 and 2017, respectively; (b, d) detail of the transect between the inner fjord and beginning of the shelf, for 2016 and 2017, respectively. Refer Figure 1, Table S5 and Methods, Sampling collection.

**
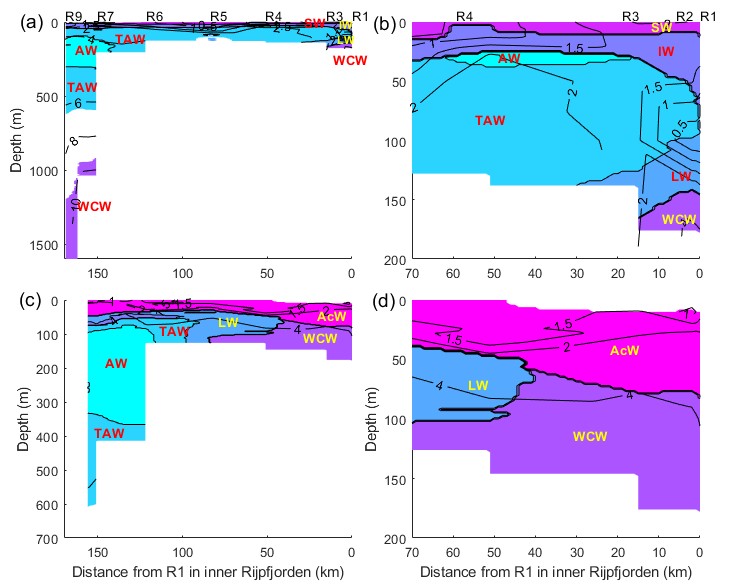
Fig. S14** Silicic acid concentration isolines (mmol m^-3^) and water masses [labels in the figure, Arctic Water (ArW), Atlantic Water (AW), Intermediate Water (IW), Local Water (LW), Surface Water (SW), Transformed Atlantic Water (TAW) and Winter-cooled Water (WCW)] along the Rijpfjorden. (a, c) the full transect for 2016 and 2017, respectively; (b, d) detail of the transect between the inner fjord and beginning of the shelf, for 2016 and 2017, respectively. Refer Figure 1, Table S5 and Methods, Sampling collection.

**
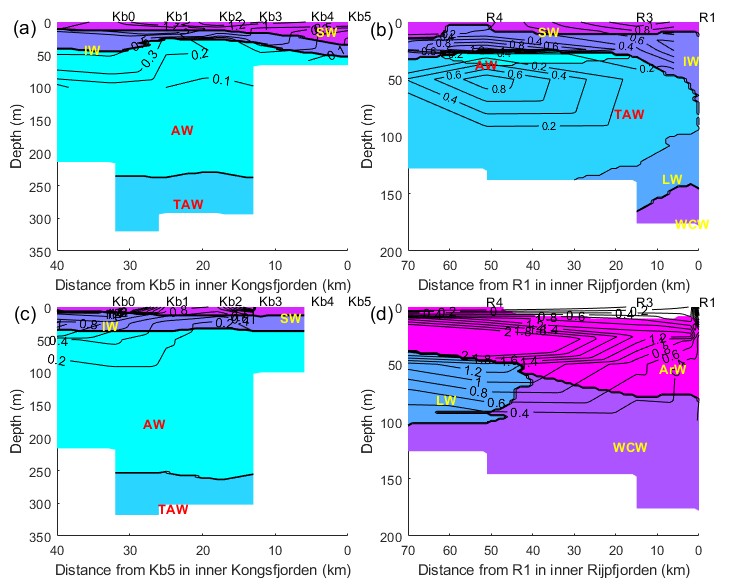
Fig. S15** Chl *a* (from bottle samples) concentration isolines (mg m^-3^) and water masses and water masses [labels in the figure, Arctic Water (ArW), Atlantic Water (AW), Intermediate Water (IW), Local Water (LW), Surface Water (SW), Transformed Atlantic Water (TAW) and Winter-cooled Water (WCW)] for 2016 (top panels) and 2017 (bottom panels). **(a)** and **(c)** Kongsfjorden transect. **(b)** and **(d)** Rijpfjorden transect. Results are shown only for the fjords and adjacent shelves (refer previous figures). Refer Figure 1, Table S5 and Methods, Sampling collection.

**
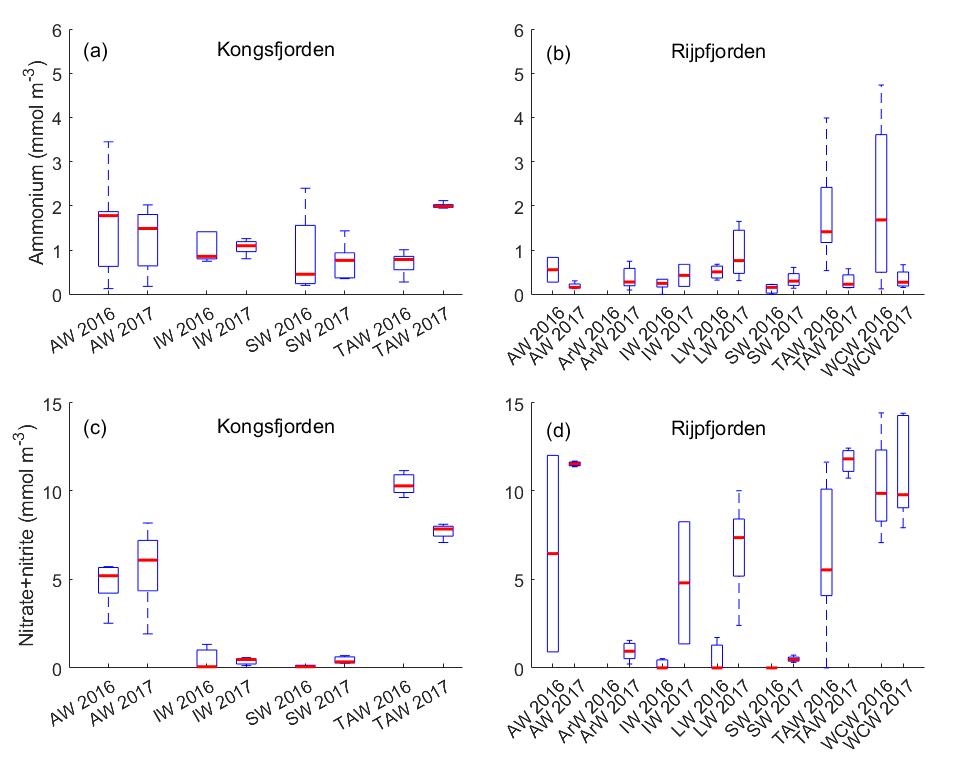
**

**Fig. S16** Box and whiskers for ammonium (a, b, Kongsfjorden and Rijpfjorden, respectively), nitrate + nitrite (c, d, Kongsfjorden and Rijpfjorden, respectively) in water masses from summers of 2016 and 2017, from fjord and shelf stations. Water masses were classified following Cottier et al. (2005): Atlantic Water (AW), Arctic Water (ArW), Intermediate Water (IW), Local Water (LW), Surface Water (SW), Transformed Atlantic Water (TAW) and Winter-cooled Water (WCW). On each box, the red line indicates the median, while the lower and upper edges of the box represent the 25th and 75th percentiles, respectively. The whiskers extend to the most extreme data points that are not considered outliers. Outliers are defined following Tukey’s rule *, as values lying more than 1.5 interquartile ranges below the 25th percentile or above the 75th percentile. (Refer Table S5 and Methods, Statistical analysis).

* Tukey, J. W.: Exploratory Data Analysis. Addison-Wesley, 1977.

**
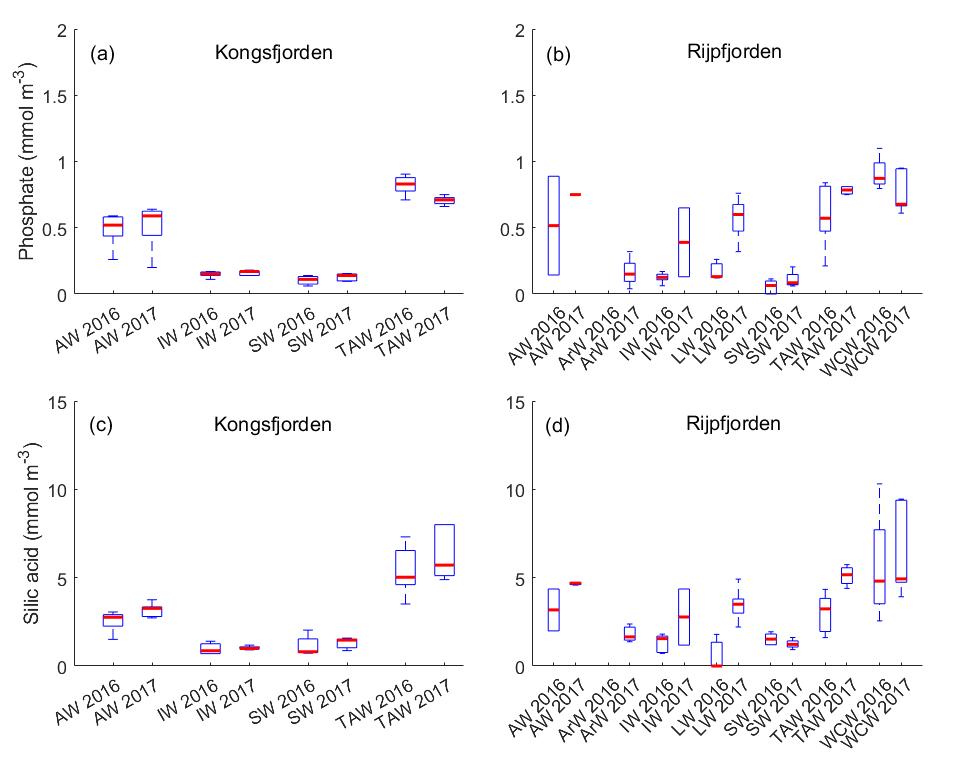
Fig. S17** The same as previous figure but for phosphate and silicic acid.


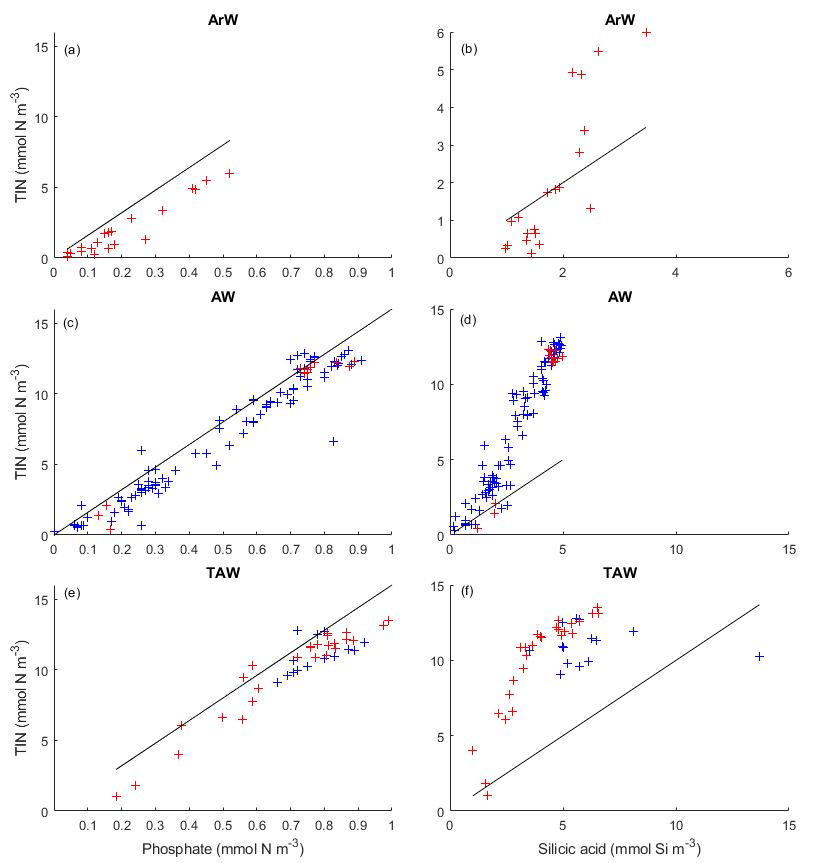


**Fig. S18** Stoichiometric ratios of: (a, b) Arctic Water (ArW), (c, d) Atlantic Water (AW) and (e, f) Transformed Atlantic Water (TAW), between Total Inorganic Nitrogen (TIN = nitrate + nitrite + ammonium) and phosphate (left panels), and TIN and silicic acid (right panels), in Kongsfjorden (blue) and Rijpfjorden (red). Black lines show the expected values assuming a Redfield molar N: P ratio of 16 (left panels) and a N: Si ratio of 1 (refer Methods, Statistical analysis and Table S5).


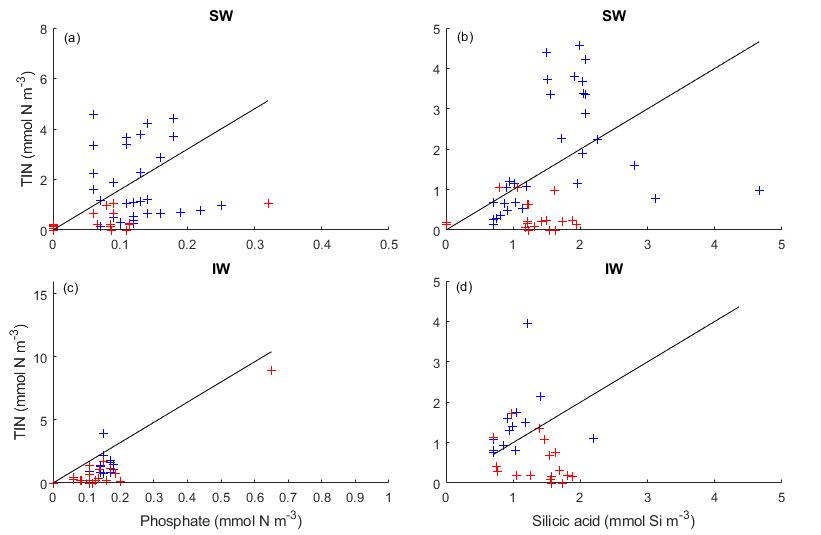


**Fig. S19** Stoichiometric ratios of: (a, b) Surface Water (SW) and (c, d) Intermediate Water (IW), between Total Inorganic Nitrogen (TIN = nitrate + nitrite + ammonium) and phosphate (left panels), and TIN and silicic acid (right panels), in Kongsfjorden (blue) and Rijpfjorden (red). Black lines show the expected values assuming a Redfield molar N: P ratio of 16 (left panels) and a N: Si ratio of 1 (refer Methods, Statistical analysis and Table S5).


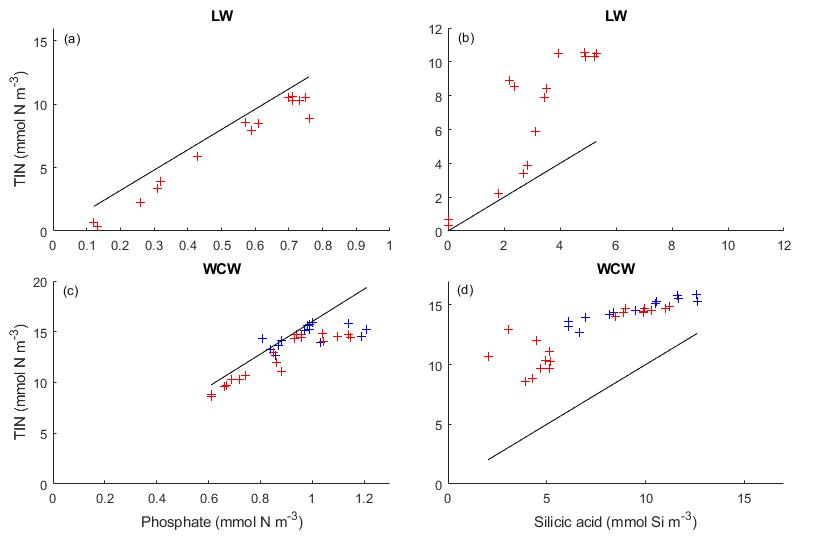


**Fig. S20** Stoichiometric ratios of: (a, b) Local Water (LW) and (c, d) Winter Cooled Water (WCW), between Total Inorganic Nitrogen (TIN = nitrate + nitrite + ammonium) and phosphate (left panels), and TIN and silicic acid (right panels), in Kongsfjorden (blue) and Rijpfjorden (red). Black lines show the expected values assuming a Redfield molar N: P ratio of 16 (left panels) and a N: Si ratio of 1 (refer Methods, Statistical analysis and Table S5).


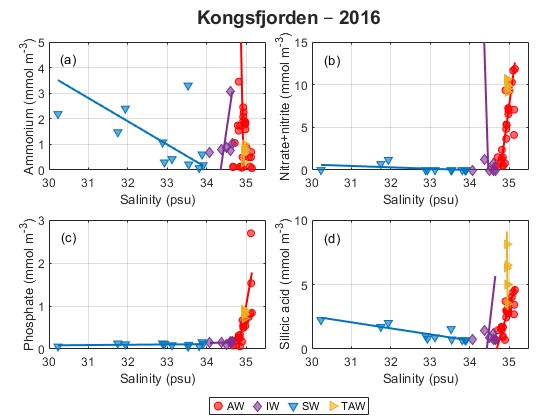


**Fig. S21** Mixing diagrams and major axis regressions between nutrients and salinity: (a) ammonium; (b) nitrate + nitrite; (c) phosphate and (d) silicic acid, for the various water masses in Kongsfjorden, using data from shelf (V10, V12 and V14) and fjord stations (Kb0 – Kb5) (Figure 1), from Summer 2016. Water masses were classified following Cottier et al. (2005): Atlantic Water (AW), Intermediate Water (IW), Surface Water (SW) and Transformed Atlantic Water (TAW) (refer Table S5 and Methods, Statistical analysis).


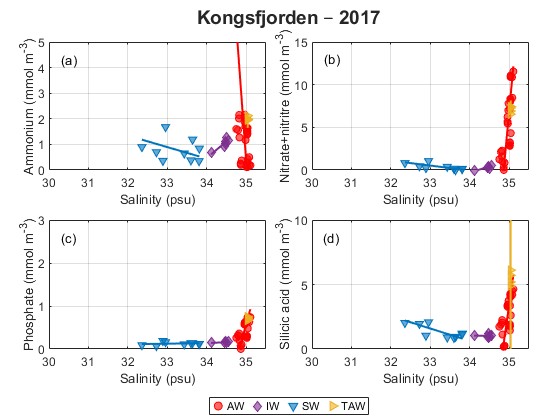


**Fig. S22** Same as previous figure but using data from Summer 2017.

**
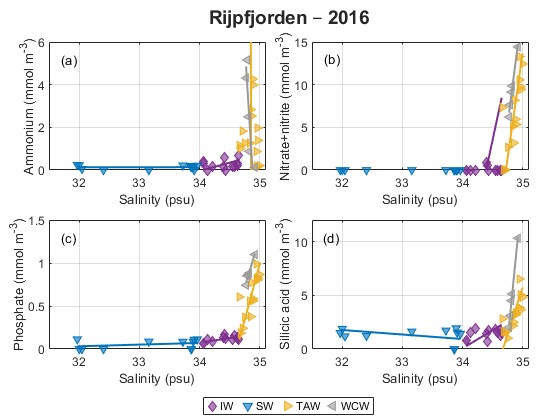
**

**Fig. S23** Mixing diagrams and major axis regressions between nutrients and salinity: (a) ammonium; (b) nitrate + nitrite; (c) phosphate and (d) silicic acid, for the various water masses in Rijpfjorden, using data from shelf (R4, R5, R6, R6-b, R7 and R7-b) and fjord stations (R1 – R3) (Figure 1), from Summer 2016. Water masses were classified following Cottier et al. (2005): Intermediate water (IW), Surface Water (SW), Transformed Atlantic Water (TAW) and Winter-cooled Water (WCW) (refer Table S5 and Methods, Statistical analysis).


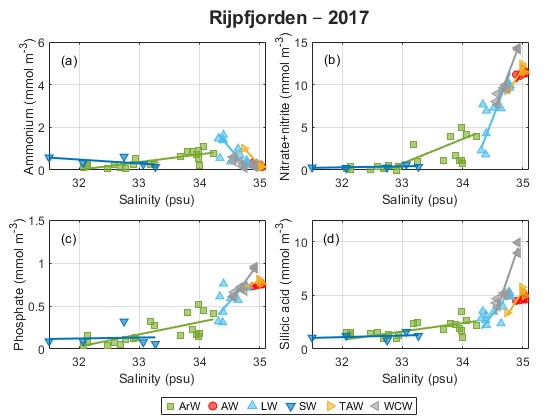


**Fig. S24** Mixing diagrams and major axis regressions between nutrients and salinity: (a) ammonium; (b) nitrate + nitrite; (c) phosphate and (d) silicic acid, for the various water masses in Rijpfjorden, using data from shelf (R4, R5, R6, R6-b, R7 and R7-b) and fjord stations (R1 – R3) (Figure 1), from Summer 2017. Water masses were classified following Cottier et al. (2005): Arctic Water (ArW), Atlantic Water (AW), Local Water (LW), Surface Water (SW), Transformed Atlantic Water (TAW) and Winter-cooled Water (WCW) (refer Table S5 and Methods, Statistical analysis).


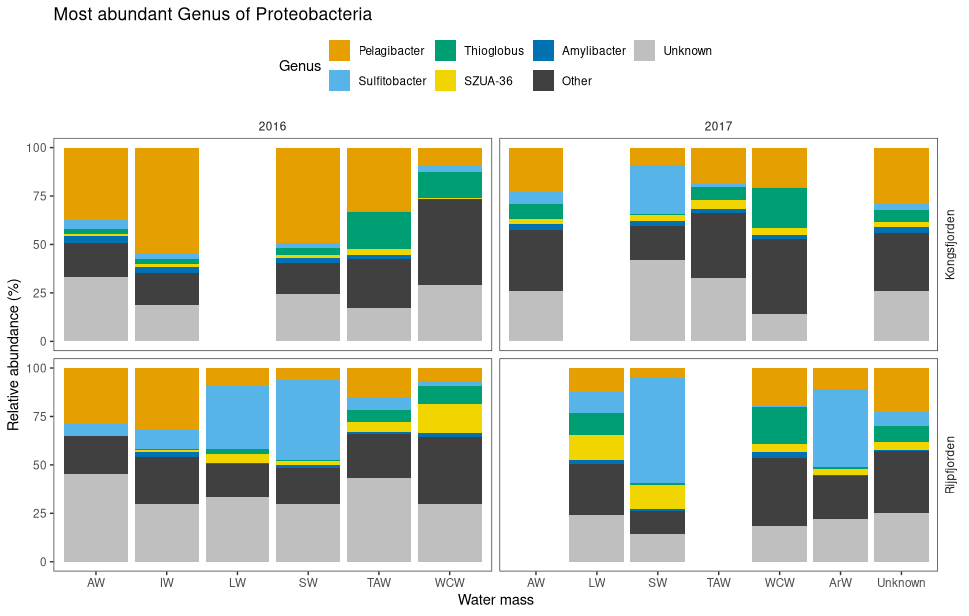


**Fig. S25** Relative abundance (%) of taxonomic groups, highlighting the most abundant genus of the *Proteobacteria* phylum. For context, the proportion of non*-Proteobacteria* was illustrated in light gray. The abscissa identifies the water masses (AW - Atlantic Water, IW - Intermediate Water, LW - Local Water, SW - Surface Water, TAW - Transformed Atlantic Water, WCW - Winter Cooled Water, ArW - Arctic Water.


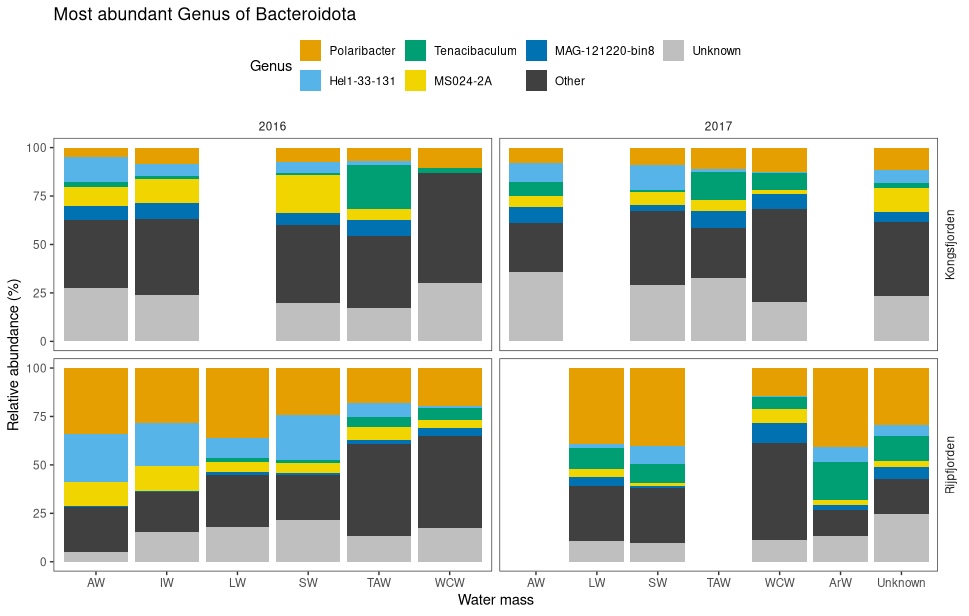


**Fig. S26** Relative abundance (%) of taxonomic groups, highlighting the most abundant genus of the Bacteroidota phylum. For context, the proportion of non-Bacteroidota was illustrated in light gray. The abscissa identifies the water masses (AW - Atlantic Water, IW - Intermediate Water, LW - Local Water, SW - Surface Water, TAW - Transformed Atlantic Water, WCW - Winter Cooled Water, ArW - Arctic Water.

**Supplementary Tables**

**Note**: Supplementary Tables S1 to S3 were made available in their original files, because they do not fit inside the document.

| **Table S4** Genes involved in the different pathways of the nitrogen cycle that were searched in samples from the present study and associated proteins. Column "Genes found" refers to the genes that were positively identified in at least one sample of the present study. | | | |
| --- | --- | --- | --- |
| **Nitrogen cycle pathway** | **Genes searched** | **Proteins** | **Genes found** |
| Nitrogen fixation | *nifD, nifK, nifH* | NifDKH | *nifH* |
|  | *anfG* | AnfG |  |
|  | *vnfD, vnfK, vnfG, vnfH* | VnfDKGH |  |
| Nitrification | *amoC, amoA, amoB* | AmoCAB | *amoA, amoC* |
|  | *hao* | Hao | *hao* |
|  | *nxrA, nxrB* | NxrAB |  |
| Dissimilatory nitrate reduction | *narG, narH, narI* | NarGHI |  |
|  | *napA, napB* | NapAB | *napA* |
|  | *nirB, nirD* | NirBD | *nirD* |
|  | *nrfA, nrfH* | NrfAH |  |
| Assimilatory nitrate reduction | *narB* | NarB | *narB* |
|  | Assimilatory nitrate reductase (NR) | NR |  |
|  | *nasA, nasB* | NasAB |  |
|  | Nitrite reductase (NIT-6) | NIT-6 |  |
|  | *nirA* | NirA |  |
|  | *nasB, nasD, nasE* | NasBDE |  |
| Denitrification | *narG, narH, narI* | NarGHI |  |
|  | *napA, napB* | NapAB |  |
|  | *nirK* | NirK | *niK* |
|  | *nirS* | NirS |  |
|  | *norB, norC* | NorBC | *norB* |
|  | *nosZ* | NosZ | *nosZ* |
| Anamox | *hzs* | Hzs |  |
|  | *hdh* | Hdh | *hdhA* |

| **Table S5** Definitions of water masses following Cottier et al. (2005). | | | |
| --- | --- | --- | --- |
|  | | | |
| **Water mass** | **Abbreviation** | **Potential temperature (ºC)** | **Salinity (psu)** |
| Arctic water | ArW | -1.5 to 1.0 | 34.30 to 34.80 |
| Atlantic water | AW | >3.0 | >34.65 |
| Intermediate water | IW | >1.0 | 34.00 to 34.65 |
| Local water | LW | -0.5 to 1.0 | 34.30 to 34.85 |
| Surface water | SW | >1.0 | <34.00 |
| Transformed Atlantic water | TAW | 1.0 to 3.0 | >34.65 |
| Winter-cooled water | WCW | <-0.5 | 34.40 to 35.00 |

**Table S6** Descriptive statistics for nutrients and Chl *a* concentrations, in different water masses [Atlantic Water (AW), Arctic Water (ArW), Intermediate Water (IW), Local Water (LW), Surface Water (SW), Transformed Atlantic Water (TAW) and Winter-cooled Water (WCW)], from fjord and shelf stations in Kongsfjorden (K) and Rijpfjorden (R), respectively, using data from summers 2016 and 2017. A dash is used when data is absent. Bold type indicates significant differences (*p* < 0.05) between fjords with the Mann-Whitney U test. We show the probability of Type I error returned from each test (*p*) and corrected (*p_corr*) with the Benjamini–Hochberg false discovery rate correction for multiple testing. Confidence limits (Low and High CL) were computed with the bootstrap method (cf. - Methods, Statistical analysis). Refer Fig. 1 for the location of the sampling stations and Table S5 for the water mass classification.

|  |  | **Sample size** | | **Medians** | | **Mean** | **Low CL** | **High CL** | **Mean** | **Low CL** | **High CL** | **Mann-Whitney** | |
| --- | --- | --- | --- | --- | --- | --- | --- | --- | --- | --- | --- | --- | --- |
| **Variable** | **Water mass** | **K** | **R** | **K** | **R** | **K** | | | **R** | | | ***p*** | ***p_corr*** |
| Ammonium | AW | 14 | 6 | 1.58 | 0.22 | 1.42 | 0.97 | 1.89 | 0.31 | 0.17 | 0.53 | **0.01** | 0.07 |
|  | ArW | 0 | 8 | - | 0.28 | - | - | - | 0.37 | 0.23 | 0.53 | - | - |
|  | IW | 10 | 8 | 0.95 | 0.25 | 1.17 | 0.88 | 1.63 | 0.31 | 0.17 | 0.47 | **0.00** | **0.00** |
|  | LW | 0 | 11 | - | 0.61 | - | - | - | 0.80 | 0.55 | 1.09 | - | - |
|  | SW | 10 | 10 | 0.61 | 0.20 | 0.83 | 0.47 | 1.29 | 0.21 | 0.12 | 0.32 | **0.00** | **0.03** |
|  | TAW | 10 | 9 | 1.48 | 0.58 | 1.36 | 0.94 | 1.76 | 1.16 | 0.50 | 2.01 | 0.18 | 0.29 |
|  | WCW | 0 | 10 | - | 0.41 | - | - | - | 1.03 | 0.32 | 2.00 | - | - |
| Nitrate+nitrite | AW | 14 | 6 | 5.66 | 11.54 | 5.27 | 4.36 | 6.13 | 9.84 | 6.23 | 11.76 | **0.02** | 0.07 |
|  | ArW | 0 | 8 | - | 0.94 | - | - | - | 1.13 | 0.63 | 1.78 | - | - |
|  | IW | 10 | 8 | 0.36 | 0.23 | 0.43 | 0.21 | 0.70 | 1.32 | 0.12 | 3.39 | 0.50 | 0.61 |
|  | LW | 0 | 11 | - | 5.92 | - | - | - | 5.10 | 3.06 | 7.08 | - | - |
|  | SW | 10 | 10 | 0.26 | 0.00 | 0.28 | 0.15 | 0.43 | 0.20 | 0.05 | 0.37 | 0.18 | 0.29 |
|  | TAW | 10 | 9 | 8.88 | 10.73 | 9.05 | 8.20 | 9.92 | 8.78 | 5.99 | 11.11 | 0.55 | 0.61 |
|  | WCW | 0 | 10 | - | 9.83 | - | - | - | 10.64 | 9.09 | 12.34 | - | - |
| Phosphate | AW | 14 | 6 | 0.54 | 0.75 | 0.52 | 0.43 | 0.60 | 0.67 | 0.45 | 0.82 | **0.05** | 0.11 |
|  | ArW | 0 | 8 | - | 0.15 | - | - | - | 0.16 | 0.11 | 0.23 | - | - |
|  | IW | 10 | 8 | 0.16 | 0.13 | 0.15 | 0.14 | 0.17 | 0.19 | 0.11 | 0.33 | 0.15 | 0.29 |
|  | LW | 0 | 11 | - | 0.52 | - | - | - | 0.46 | 0.33 | 0.59 | - | - |
|  | SW | 10 | 10 | 0.12 | 0.08 | 0.12 | 0.10 | 0.13 | 0.08 | 0.04 | 0.11 | **0.05** | 0.11 |
|  | TAW | 10 | 9 | 0.74 | 0.76 | 0.76 | 0.72 | 0.82 | 0.68 | 0.54 | 0.79 | 0.73 | 0.73 |
|  | WCW | 0 | 10 | - | 0.83 | - | - | - | 0.82 | 0.72 | 0.91 | - | - |
| Silicic acid | AW | 14 | 6 | 2.88 | 4.61 | 2.72 | 2.30 | 3.08 | 4.18 | 3.28 | 4.69 | **0.01** | 0.07 |
|  | ArW | 0 | 8 | - | 1.65 | - | - | - | 1.80 | 1.55 | 2.06 | - | - |
|  | IW | 10 | 8 | 0.99 | 1.55 | 1.00 | 0.87 | 1.13 | 1.70 | 1.10 | 2.55 | **0.05** | 0.11 |
|  | LW | 0 | 11 | - | 3.14 | - | - | - | 2.68 | 1.78 | 3.52 | - | - |
|  | SW | 10 | 10 | 1.23 | 1.34 | 1.22 | 0.97 | 1.48 | 1.30 | 0.95 | 1.59 | 0.43 | 0.58 |
|  | TAW | 10 | 9 | 5.45 | 4.34 | 6.27 | 4.98 | 8.14 | 3.93 | 3.01 | 4.78 | **0.02** | 0.07 |
|  | WCW | 0 | 10 | - | 4.94 | - | - | - | 5.98 | 4.49 | 7.64 | - | - |
| Chl a | AW | 14 | 5 | 0.17 | 0.17 | 0.34 | 0.19 | 0.52 | 0.26 | 0.10 | 0.49 | 0.67 | 0.71 |
|  | ArW | 0 | 8 | - | 1.61 | - | - | - | 2.18 | 1.16 | 3.57 | - | - |
|  | IW | 10 | 8 | 0.30 | 0.40 | 0.41 | 0.23 | 0.60 | 0.72 | 0.28 | 1.37 | 0.41 | 0.58 |
|  | LW | 0 | 11 | - | 0.38 | - | - | - | 0.96 | 0.30 | 2.13 | - | - |
|  | SW | 10 | 10 | 0.90 | 0.73 | 1.04 | 0.68 | 1.48 | 0.79 | 0.46 | 1.14 | 0.52 | 0.61 |
|  | TAW | 0 | 6 | - | 0.15 | - | - | - | 0.41 | 0.10 | 0.86 | - | - |
|  | WCW | 0 | 3 | - | 0.26 | - | - | - | 0.31 | 0.24 | 0.44 | - | - |

**Table S7** Significant major axis regressions between nutrient concentrations and salinity, calculated for each year, each fjord and each water mass. We show sample size (n), the slope and the probability of Type I error returned from each regression (*p*) and corrected (*p_corr*) with the Benjamini–Hochberg false discovery rate correction for multiple testing (refer Methods, Statistical analysis and Figs S21-S24).

| **Nutrient** | **Fjord** | **Year** | **WaterMass** | **n** | **Slope** | ***p*** | ***p_corr*** |
| --- | --- | --- | --- | --- | --- | --- | --- |
| Ammonium | Kongsfjorden | 2016 | TAW | 6 | 32.845 | 0.0094 | 0.0279 |
|  | Rijpfjorden | 2017 | ArW | 20 | 0.363 | 0.0001 | 0.0004 |
|  | Rijpfjorden | 2017 | LW | 12 | -3.483 | 0.0002 | 0.0008 |
|  | Rijpfjorden | 2017 | WCW | 10 | -1.384 | 0.0099 | 0.0283 |
| Nitrate+nitrite | Kongsfjorden | 2016 | AW | 25 | 34.309 | 0 | 0 |
|  | Kongsfjorden | 2016 | TAW | 6 | -77.418 | 0.0029 | 0.011 |
|  | Kongsfjorden | 2017 | AW | 28 | 49.664 | 0 | 0 |
|  | Kongsfjorden | 2017 | SW | 9 | -0.581 | 0.0155 | 0.0407 |
|  | Rijpfjorden | 2016 | TAW | 14 | 49.168 | 0 | 0.0001 |
|  | Rijpfjorden | 2017 | ArW | 20 | 2.864 | 0.0001 | 0.0008 |
|  | Rijpfjorden | 2017 | LW | 12 | 18.497 | 0.0002 | 0.0008 |
|  | Rijpfjorden | 2017 | WCW | 10 | 18.5 | 0 | 0 |
| Phosphate | Kongsfjorden | 2017 | AW | 28 | 3.323 | 0 | 0.0001 |
|  | Rijpfjorden | 2016 | IW | 12 | 0.102 | 0.0126 | 0.0345 |
|  | Rijpfjorden | 2016 | TAW | 14 | 2.621 | 0 | 0.0001 |
|  | Rijpfjorden | 2017 | ArW | 20 | 0.142 | 0.0007 | 0.003 |
|  | Rijpfjorden | 2017 | AW | 7 | 0.192 | 0.0085 | 0.0262 |
|  | Rijpfjorden | 2017 | LW | 12 | 0.786 | 0.0045 | 0.0151 |
|  | Rijpfjorden | 2017 | WCW | 10 | 0.964 | 0 | 0 |
| Silicic acid | Kongsfjorden | 2016 | AW | 25 | 10.434 | 0 | 0 |
|  | Kongsfjorden | 2016 | SW | 11 | -0.469 | 0.0014 | 0.0056 |
|  | Kongsfjorden | 2017 | AW | 28 | 21.564 | 0 | 0 |
|  | Kongsfjorden | 2017 | SW | 9 | -0.967 | 0.0084 | 0.0262 |
|  | Rijpfjorden | 2016 | TAW | 14 | 17.564 | 0.0036 | 0.0128 |
|  | Rijpfjorden | 2016 | WCW | 5 | 56.227 | 0.0006 | 0.0026 |
|  | Rijpfjorden | 2017 | ArW | 20 | 0.787 | 0.019 | 0.0483 |
|  | Rijpfjorden | 2017 | LW | 12 | 7.261 | 0.0006 | 0.0026 |
|  | Rijpfjorden | 2017 | WCW | 10 | 16.609 | 0 | 0.0001 |

**Table S8** Biogeochemical ammonium *Sources-Sinks* for Kongsfjorden and Rijpfjorden in 2016 and 2017, estimated using equation 1, based on differences of average salinities and concentrations of nitrate + nitrite found in the shelf stations in endmember water masses and in fjord stations in “diluted” water masses. The following shelf stations were considered for Kongsfjorden and Rijpfjorden, respectively: V10, V12, V14, and R4, R5, R6, R6-b, R7 and R7-b. The following fjord stations were considered for Kongsfjorden and Rijpfjorden, respectively: Kb1-Kb5 and R1-R3. The same water masses were used to compute differences between values in the shelf and values in the fjord, in *both* years, in the case of Kongsfjorden: AWs (Atlantic Water in the shelf) versus IW, SW or AWf (Atlantic Water in the fjord). In the case of Rijpfjorden, different water masses were used in 2016 and in 2017, due to major differences in the hydrographic conditions. Therefore, in 2016, we computed differences in salinity and concentration of nitrate + nitrite for TAWs (Transformed Atlantic Water in the shelf) *versus* IW, SW or TAWf (Transformed Atlantic Water in the fjord), whereas in the 2017, we computed differences for ArWs (Arctic Water in the shelf) *versus* ArWf (Arctic Water in the fjord) and, LWs (Local Water in the shelf) *versus* LWf (Local Water in the fjord). In all cases, when water masses are indicated without “s” or “f” to specify shelf and fjord values, they refer to fjord values only. Calculations were restricted to the top 100 m and assumed zero salinities and nitrate + nitrite concentrations for freshwater endmembers. See Methods, Statistical analysis for details and Figure 1 for the locations of shelf and fjord stations.

| **Ammonium *Sources-Sinks* (μmol kg^-1^)** | | | | | | | |
| --- | --- | --- | --- | --- | --- | --- | --- |
| **Kongsfjorden** | | | | | **Rijpfjorden** | | |
| **Year** | **AWs *versus* IW** | | **AWs *versus* SW** | **AWs *versus* AWf** | **TAWs *versus* IW** | **TAWs *versus* SW** | **TAWs *versus* TAWf** |
| **2016** | 0.9 | | 1.0 | 1.8 | -1.6 | -1.7 | -0.5 |
|  | |  |  |  | **ArWs *versus* ArWf** | | |
| **2017** | 0.7 | | 0.5 | 1.4 | 0.3 | | |

**Table S9** As Table S7 but for phosphate.

|  | **Phosphate *Sources-Sinks* (μmol kg^-1^)** | | | | | | |
| --- | --- | --- | --- | --- | --- | --- | --- |
| **Kongsfjorden** | | | | | **Rijpfjorden** | | |
| **Year** | **AWs *versus* IW** | | **AWs *versus* SW** | **AWs *versus* AWf** | **TAWs *versus* IW** | **TAWs *versus* SW** | **TAWs *versus* TAWf** |
| **2016** | -0.5 | | -0.6 | -0.3 | -0.5 | -0.5 | -0.4 |
|  | |  |  |  | **ArWs *versus* ArWf** | | |
| **2017** | -0.1 | | -0.2 | 0.1 | -0.1 | | |

**Table S10** As Table S8 but for silicic acid.

|  | **Silic acid *Sources-Sinks* (μmol kg^-1^)** | | | | | | |
| --- | --- | --- | --- | --- | --- | --- | --- |
| **Assuming zero silicic acid concentration in freshwater endmember** | | | | | | | |
| **Kongsfjorden** | | | | | **Rijpfjorden** | | |
| **Year** | **AWs *versus* IW** | | **AWs *versus* SW** | **AWs *versus* AWf** | **TAWs *versus* IW** | **TAWs *versus* SW** | **TAWs *versus* TAWf** |
| **2016** | -1.6 | | -1.1 | -0.4 | -1.1 | -0.9 | -1.1 |
|  | |  |  |  | **ArWs *versus* ArWf** | | |
| **2017** | -0.8 | | -0.4 | 0.8 | 0.0 | | |

**Table S11** PERMANOVA results for the effects of water mass, year, transect and depth on procaryotic community composition, accounting for all variable interactions (denoted by “:”). 999 permutations were used. The formula used is indicated below the table, where “y” denotes community composition.

| **Variables** | **Df** | **SS** | **R^2^** | **F** | **p-value** | **Significance** |
| --- | --- | --- | --- | --- | --- | --- |
| water mass | 8 | 4.3060 | 0.26650 | 3.7456 | 0.001 | *** |
| year | 1 | 1.0833 | 0.06705 | 7.5386 | 0.001 | *** |
| transect | 1 | 1.0981 | 0.06796 | 7.6412 | 0.001 | *** |
| depth | 3 | 1.2345 | 0.07640 | 2.8636 | 0.001 | *** |
| water mass:year | 4 | 0.8311 | 0.05143 | 1.4458 | 0.030 | * |
| water mass:transect | 5 | 1.5459 | 0.09567 | 2.1515 | 0.001 | *** |
| water mass:depth | 10 | 1.8913 | 0.11705 | 1.3161 | 0.029 | * |
| Residual | 29 | 4.1674 | 0.25792 |  |  |  |
| Total | 61 | 16.1576 | 1.00000 |  |  |  |
| formula: y ~ (year + transect + depth)*water mass | | | | | | |

**Table S12** PERMANOVA results about the effects of nutrients, salinity, temperature and transect on procaryotic community composition. 999 permutations were used. The formula used is indicated below the table, where “y” denotes community composition. Variable interactions with transect were not possible to test due to insufficient data.

| **Variables** | **Df** | **SS** | **R^2^** | **F** | **p-value** | **Significance** |
| --- | --- | --- | --- | --- | --- | --- |
| NH4 | 1 | 0.3052 | 0.06312 | 1.854 | 0.044 | * |
| NO2 | 1 | 0.4613 | 0.09540 | 2.802 | 0.008 | ** |
| NO3 | 1 | 0.3608 | 0.07462 | 2.191 | 0.034 | * |
| PO4 | 1 | 0.1819 | 0.03762 | 1.105 | 0.328 |  |
| Si | 1 | 0.2101 | 0.04344 | 1.176 | 0.238 |  |
| temperature | 1 | 0.3190 | 0.06597 | 1.938 | 0.045 | * |
| salinity | 1 | 0.4064 | 0.08404 | 2.469 | 0.008 | ** |
| transect | 1 | 0.2890 | 0.05978 | 1.756 | 0.064 |  |
| Residual | 13 | 2.3016 | 0.44256 |  |  |  |
| Total | 22 | 4.8352 | 1 |  |  |  |
| formula: y ~ NH4 + NO2 + NO3 + PO4 + Si + temperature + salinity + transect | | | | | | |

**Table S13** PERMANOVA results about the effects of nutrients, salinity, and temperature on procaryotic community composition, accounting for the effect of year. 999 permutations were used. Interactions were denoted by ":". The formula used is indicated below the table, where “y” denotes community composition.

| **Variable** | **Df** | **SS** | **R^2^** | **F** | **p-value** | **Significance** |
| --- | --- | --- | --- | --- | --- | --- |
| NH4 | 1 | 0.3052 | 0.06312 | 3.0368 | 0.005 | ****** |
| NO2 | 1 | 0.4613 | 0.0954 | 4.59 | 0.001 | ******* |
| NO3 | 1 | 0.3608 | 0.07462 | 3.5902 | 0.004 | ****** |
| PO4 | 1 | 0.1819 | 0.03762 | 1.8102 | 0.067 |  |
| Si | 1 | 0.2101 | 0.04344 | 2.0903 | 0.034 | ***** |
| temperature | 1 | 0.319 | 0.06597 | 3.1743 | 0.002 | ****** |
| salinity | 1 | 0.4064 | 0.08404 | 4.0435 | 0.001 | ******* |
| year | 1 | 0.8133 | 0.16821 | 8.0932 | 0.001 | ******* |
| NH4:year | 1 | 0.1329 | 0.02749 | 1.3227 | 0.206 |  |
| NO2:year | 1 | 0.1841 | 0.03807 | 1.8317 | 0.077 |  |
| NO3:year | 1 | 0.3255 | 0.06731 | 3.2387 | 0.004 | ****** |
| PO4:year | 1 | 0.0695 | 0.01437 | 0.6916 | 0.757 |  |
| Si:year | 1 | 0.1231 | 0.02547 | 1.2253 | 0.29 |  |
| temperature:year | 1 | 0.1198 | 0.02477 | 1.1918 | 0.278 |  |
| salinity:year | 1 | 0.119 | 0.0246 | 1.1837 | 0.298 |  |
| Residual | 7 | 0.7035 | 0.14549 |  |  |  |
| Total | 22 | 4.8352 | 1 |  |  |  |
| formula: y ~ (NH4 + NO2 + NO3 + PO4 + Si + temperature + salinity)*year | | | | | | |
